# Metal ion-amplified phototherapy for tumors: Mechanisms, nanomaterial design, and synergistic strategies

**DOI:** 10.1016/j.mtbio.2026.103039

**Published:** 2026-03-17

**Authors:** Yang Chen, Yehui Kang, Lichen Ji, Liya Yu, Longcai Liu, Xiaozhou Mou, Yu Cai

**Affiliations:** Centre for Rehabilitation Medicine, Rehabilitation & Sports Medicine Research Institute of Zhejiang Province, Department of Rehabilitation Medicine, Cancer Centre, Zhejiang Provincial People's Hospital (Affiliated People's Hospital), Hangzhou Medical College, Hangzhou, Zhejiang, 310014, China

**Keywords:** Metal ion-dependent cell death, Phototherapy, Synergistic therapy, Nanomaterials, Cancer treatment

## Abstract

Cancer remains a major threat to the health of human for its high incidences and mortality. Traditional treatment methods, such as surgery, radiotherapy and chemotherapy, are often limited by serious side effects and insufficient curative effect. Phototherapy, including photodynamic therapy (PDT) and photothermal therapy (PTT), has become a promising alternative therapy. Although PDT and PTT exhibit intrinsic tumor selectivity, collateral damage to surrounding normal tissues may PDT has strong tumor selectivity still occur under certain conditions, particularly due to light scattering, heat diffusion, or off-target photosensitizer accumulation. Recent progress suggests that metal ion-dependent cell death (MIDCD), including ferroptosis, cuproptosis, and others, can be strategically integrated with phototherapy to partially mitigate these limitations or provide alternative therapeutic routes under challenging tumor microenvironmental conditions. This review systematically discusses the mechanisms and synergistic effects of the combination of PDT/PTT and metal ion interference therapies, which emphasizes the roles of iron, copper, calcium, zinc, magnesium and manganese in enhancing the treatment efficacy. We also summarize the design of metal-based nanomaterials and inducers, which made the spatiotemporal controlled ion release and multimodal therapy synergistic. Finally, we discuss the challenges of clinical transformation and future directions, and emphasized the potential of metal ion amplified phototherapy as an effective strategy for accurate cancer treatment.

## Graphical abstract

**Figure:**
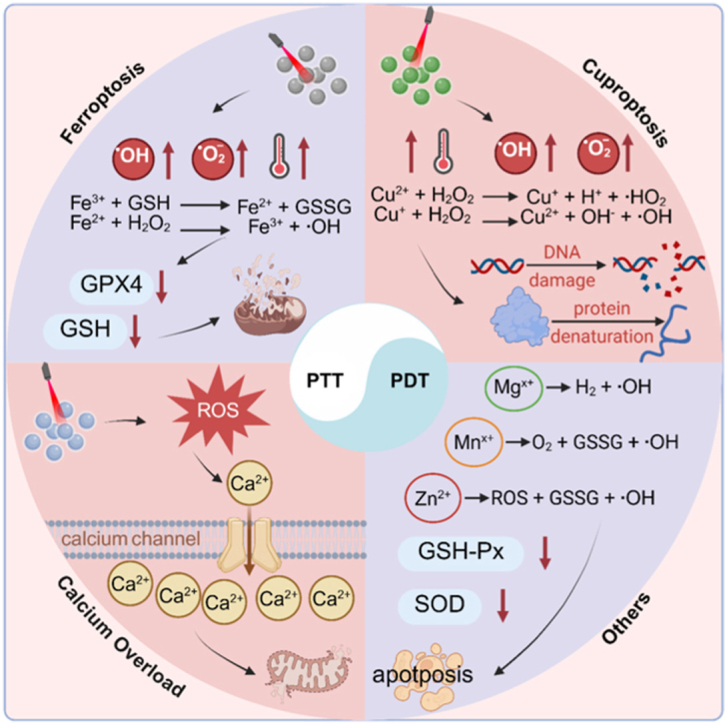

## 1 Introduction

With its rising incidence and high mortality rates, cancer has emerged as a major threat to human health. Conventional treatments, including surgery, chemotherapy, radiotherapy, and immunotherapy, remain the clinical gold standards and have achieved substantial therapeutic success. However, their efficacy can be compromised in certain cases by tumor heterogeneity, therapeutic resistance, or treatment associated adverse effects, motivating the exploration of complementary and synergistic strategies [[1], [2], [3], [4]]. In addition, these treatments will cause the immune system disorder of patients, prevent the rapid elimination of new and residual cancer cells, and lead to tumors, metastases, metastases and diseases [[5], [6], [7], [8]]. With the increasing number of deaths from diseases and cancers, it is still the focus of basic medical research to formulate new treatment strategies [[9], [10], [11], [12]]. The current research methods include immunotherapy, PTT, PDT, chemodynamic therapy (CDT), and sonodynamic therapy (SDT), that have garnered significant attention [13]. As emerging approaches in oncology, PDT and PTT employ distinct mechanisms for tumor eradication. PDT induces tumor cell death through photochemical reactions, and PTT utilizes the photothermal effect to ablate tumors by converting light energy into heat [[14], [15], [16], [17]]. In terms of core principles and key drugs, PDT must be given before operation to accumulate photosensitive substances (PSs) in tumor tissues, and then irradiated with light with a specific wavelength to generate reactive oxygen species (ROS) that induce oxidative damage and subsequent tumor cell death [18,19]. The effect is mild and highly selective, working only where all three elements are present, including PS, light, and oxygen. PTT can absorb near infrared (NIR) light source and convert it into heat. High temperature destroys the cell structure [20,21]. Quick and effective in the absence of oxygen [22]. PDT is generally considered to exhibit relatively high tumor selectivity owing to localized light activation and preferential photosensitizer accumulation. However, this selectivity is not absolute and can be compromised in complex TME or under high irradiation doses [23,24]. It does not induce drug resistance, can be used multiple times, and can also destroy tumor blood vessels, inhibit metastasis, and activate local immunity [25,26]. PTT has a short treatment cycle and simple operation, and NIR light can penetrate several centimeters deep, making it suitable for some deep-seated tumors [25,27]. However, studies have proven that there are always many problems with a single treatment method. For example, PDT is oxygen dependent and less effective against hypoxic tumors, such as the centers of late-stage solid tumors [28,29]. PSs may cause photosensitive skin reactions, and the light source typically has a shallow penetration depth, making it suitable only for superficial or minimally invasive accessible tumors [[30], [31], [32]]. However, high temperature PTT will burn the skin, and low temperature PTT may greatly reduce the tumor killing rate [[33], [34], [35]]. Therefore, an increasing number of studies are combining PDT and PTT with other methods for treatment.

MIDCD is a prominent form of nonapoptotic cell death. It is defined as cell death triggered by an imbalance of specific intracellular metal ions and mediated by specific molecular pathways [[36], [37], [38]]. MIDCD is primarily defined as regulated cell death modalities in which metal ions directly act as indispensable execution factors. Metal ions that predominantly function as signaling modulators, such as Ca^2+^, are discussed separately as synergistic stress amplifiers rather than canonical MIDCD subtypes [[39], [40], [41]]. It is distinct from classic cell death types such as apoptosis and pyroptosis, and it does not rely on apoptosis related proteins or exhibit typical apoptotic morphology, such as the formation of apoptotic bodies [42,43]. Under electron microscopy, it often manifests as early disruption of cell membrane integrity and swelling of organelles [44,45]. Essentially, MIDCD is a result of cellular regulatory failure of metal ions. When metal transporters are abnormal or metallochaperone proteins malfunction, MIDCD may be induced [46,47]. Biologically, MIDCD can clearly damage cells with metal ion accumulation and restrict pathogen infection to maintain tissue homeostasis [48,49]. It is also associated with various diseases and has become a new target for cancer therapy [50]. Therapies that disrupt the metal ion homeostasis of cancer cells and specifically induce MIDCD have entered some clinical trial stages [51,52].

**Fig. 1:**
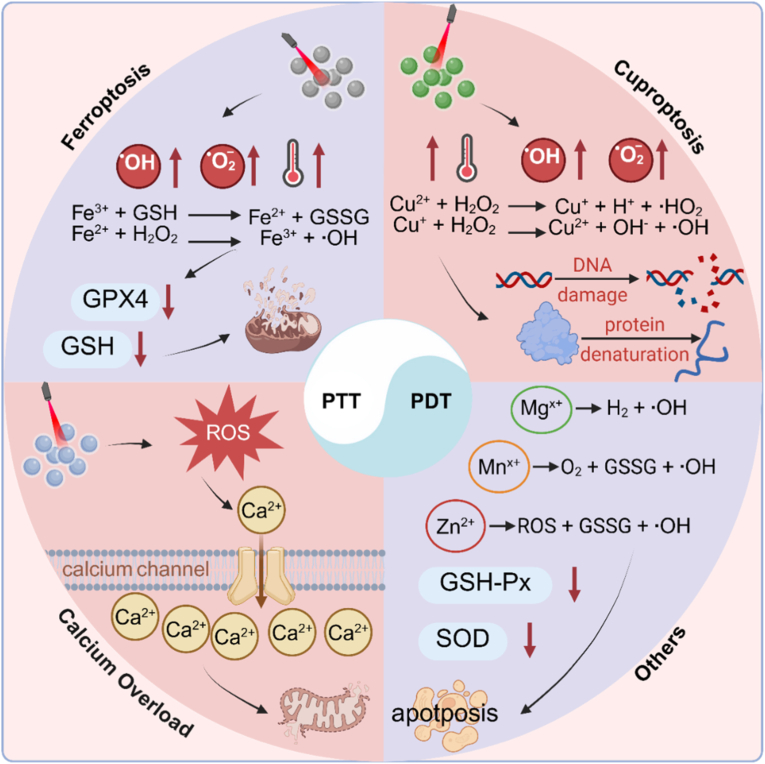
Schematic diagram of synergetic MIDCD and phototherapy for enhanced anticancer treatment. The schematic depicts the rational design and therapeutic mechanism of nanomaterials that integrate phototherapy with controlled metal ion regulation. Upon tumor accumulation, the nanoplatforms respond to intrinsic tumor microenvironment (TME) cues or external light irradiation, triggering localized release of metal ions such as Fe^2+^, Cu^+^, or Ca^2+^. Photodynamic or photothermal activation generates ROS or hyperthermia, which synergistically amplifies metal ion-mediated redox reactions, mitochondrial dysfunction, and ion-overload. These interconnected processes collectively drive MIDCD, leading to enhanced tumor cell eradication. Created with BioRender.com.

This review systematically interweaves the mechanistic foundations of PTT, PDT, and MIDCD, framing their integration as a coherent and mutually reinforcing multimodal strategy for cancer therapy rather than parallel or additive interventions. By following advances in nanomaterial engineering, it becomes evident that rationally designed platforms can seamlessly couple the PTT/PDT with the catalytic activity and signaling interference intrinsic to MIDCD, thereby initiating a cascade of interconnected biological events that extend beyond the cytotoxic ceiling achievable by phototherapy alone. As shown in Fig. 1, within this framework, precisely engineered nanostructures assume a central role by enabling spatiotemporally regulated liberation of metal ions such as Fe, Cu, Ca, Mn, and Zn in response to tumor microenvironmental cues or external light irradiation, which in turn exacerbates oxidative stress, disrupts mitochondrial homeostasis, and drives metabolic collapse across multiple regulatory layers. Mechanistically, this integration is sustained by a bidirectional reinforcement process in which phototherapy induced thermal and oxidative perturbations accelerate metal ion release, redox cycling, and intracellular redistribution, thereby intensifying metal dependent lethal pathways including lipid peroxidation (LPO) and organelle dysfunction, while concomitantly, MIDCD associated breakdown of redox balance and cellular resilience lowers the tolerance threshold to photothermal damage or photoinduced reactive species. Such reciprocal amplification establishes a positive feedback loop, positioning phototherapy as an external trigger and metal ions as intracellular signal and damage amplifiers. As metal ion augmented phototherapeutic systems continue to evolve, their functional scope naturally expands toward imaging guided therapy, enhancement of immunogenic cell death, and considerations central to translational development, thereby motivating a forward-looking discussion on emerging design strategies aimed at overcoming current limitations and accelerating the transition from conceptually synergistic constructs to clinically impactful, collaborative cancer therapeutics.

## 2 Mechanisms of phototherapy

**Fig. 2:**
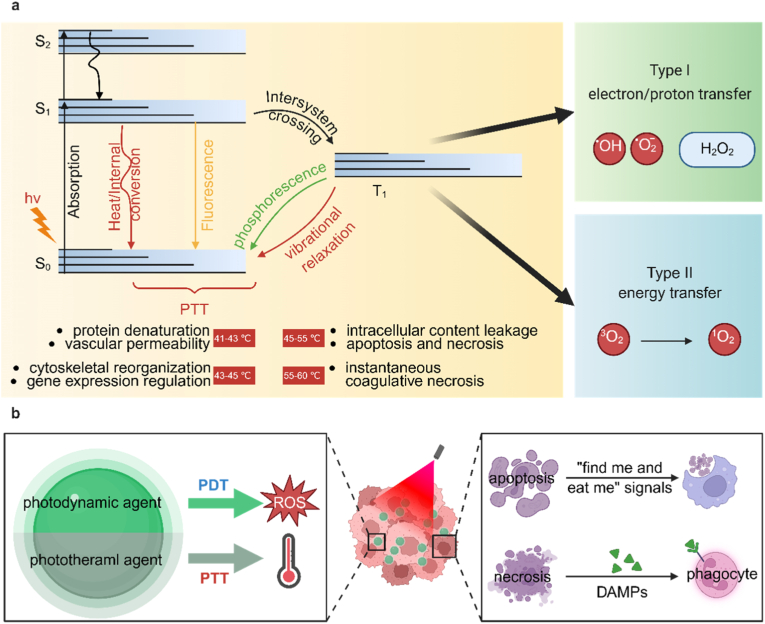
(a) Photosensitization Processes Illustrated by a Modified Jablonski Diagram. Light exposure takes a photosensitizer molecule from the ground singlet state (S_0_) to an excited singlet state (S_1_). The molecule in S_1_ may undergo intersystem crossing to an excited triplet state (T_1_) and then either form radicals via a Type I reaction or, more likely, transfer its energy to molecular oxygen (^3^O_2_) and form ^1^O_2_, which is the major cytotoxic agent involved in PDT. (b) The mechanisms by which PTT or PDT induce apoptosis in tumor cells. PDT and PTT can both initiate antitumor immune responses through the immunogenic cell death (ICD) mechanism. This process involves the release of a series of DAMPs and cytokines, which promote the recruitment and maturation of antigen-presenting cells (APCs), cross presentation, and phagocytosis. Tumor antigens are then presented to T cells, ultimately activating the antitumor immune response. Created with BioRender.com.

Phototherapy utilizes light with a certain wavelength to irradiate the photosensitizer in the tumor sites [53,54]. As shown in Fig. 2A, based on the mechanism of inducing cancer cell death, it can be divided into PDT and PTT [55]. In Fig. 2B, PDT process begins with the injection of a PS that selectively accumulates in tumors. Upon irradiation with the appropriate light wavelength, the accumulated PS initiates photochemical reactions to produce ROS, ultimately causing irreversible cellular destruction and therapeutic tumor ablation [38,56,57]. ROS is mainly derived from the superoxide anion and free radical formed by the transfer of electrons to molecular oxygen or other electron receptors when the photosensitizer is excited by light of specific wavelength (Type I reaction), or the singlet oxygen (^1^O_2_) generated by the transfer of electronic energy to ground state molecular oxygen (Type II reaction) [13,58]. Although many PSs have been synthesized, due to the typical hypoxia in TME, the production of ROS is low, which greatly hinders its therapeutic effect [59]. PTT is the other minimally invasive treatment approach that utilizes photothermal agents to convert light energy into heat, selectively destroying tumor cells while sparing the surrounding healthy tissues [60,61]. It has gained significant attention as a promising modality for cancer treatment due to its ability to provide localized and targeted therapy [62].

Traditional light sources has limited penetration to tissues, which hinders the treatment of deep tumors [63]. To solve this problem, NIR-II (900-1700 nm) phototherapy came into being, extending penetration depth to 3 cm [64,65]. When combined with nano delivery systems to enhance tumor targeting, it can significantly improve the therapeutic effect of deep tissue [66,67]. Moreover, hypoxia in tumors restricts PDT effectiveness [68]. Innovative strategies like self-oxygenating nanoplatforms can generate oxygen in situ or normalize tumor vasculature to increase oxygen supply [69,70]. PTT may also have the risk of damaging healthy tissues by thermal diffusion, while PDT causes skin photosensitivity [71,72]. Intelligent response nanosystem can switch treatment modes according to TME clues, keep “off” in the circulation, activate PTT and PDT when the tumor arrives, and release chemotherapeutic agents for multimode precise treatment [73]. The future research will focus on the development of multifunctional photosensitizers, optical switching systems and immunotherapy [74].

## 3 Metal ion-dependent cell death

While these general design principles provide a unified framework, the biological outcomes of metal ion amplified phototherapy are highly dependent on the specific metal ions involved. Accordingly, the next sections systematically discuss representative metal ions and their distinct mechanistic contributions to MIDCD.

### 3.1 Ferroptosis

Ferroptosis is a recently discovered regulated form of cell death, which is different from other well-known cell death mechanisms, such as apoptosis and necrosis [75]. It is characterized by iron-dependent accumulation of lipid peroxides, which leads to oxidative damage and subsequent cell death [76]. Ferroptosis is considered as a potential therapeutic target for various diseases, including cancer, neurodegenerative diseases and ischemia reperfusion injury [77,78]. In the context of cancer, inducing ferroptosis in tumor cells can offer a novel approach to selectively eliminate cancer cells while sparing normal cells [79]. The iron dependency and LPO characteristic of ferroptosis provide opportunities for developing targeted therapeutic strategies aimed at triggering or inhibiting this regulated cell death pathway [80]. So, ferroptosis is an iron dependent form of regulated cell death characterized by the accumulation of lipid peroxides and subsequent oxidative damage.

#### 3.1.1 Mechanisms of ferroptosis

**Fig. 3:**
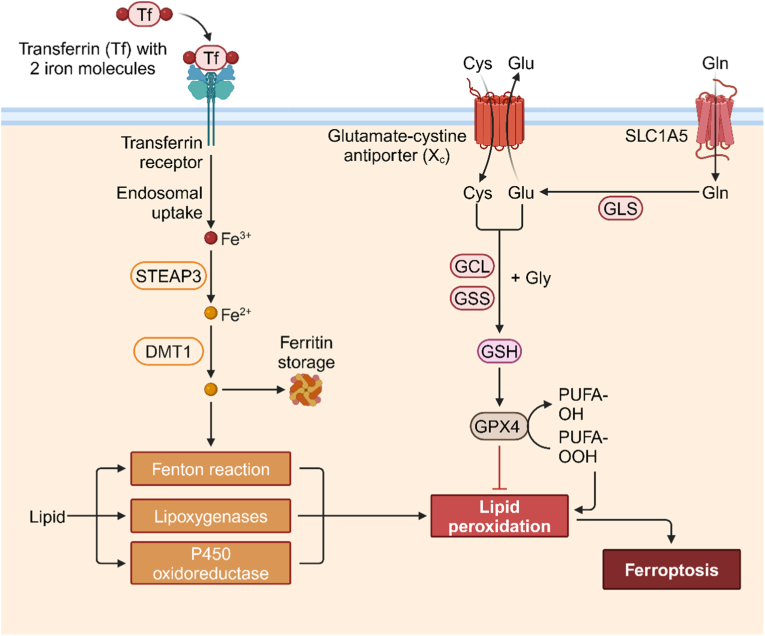
Mechanism of ferroptosis: ferroptosis is a kind of programmed cell death that depends on iron. Its mechanisms mainly include the accumulation of iron ions and their effect, LPO, the antioxidant system not being balanced, and the control of signaling pathways. Created with BioRender.com.

Ferroptosis involves a complicated interaction of molecular pathways and key factors that regulate the process (Fig. 3) [81]. Three important parts of ferroptosis are iron metabolism, LPO, and the glutathione (GSH) system, and it includes the enzyme GPX4. Iron plays a very important role in regulating ferroptosis [82,83]. Cellular iron levels are strictly controlled by proteins that take part in iron uptake, storage, and export [84,85]. Iron import is mainly carried out by transferrin receptor 1 (TFR1) and divalent metal transporter 1 (DMT1), while iron export is helped by ferroportin. When these iron transporters are not regulated properly, they can break iron homeostasis and lead to ferroptosis being easily affected [86]. And iron is also a key part in the Fenton reaction, and this reaction makes highly reactive •OH from H_2_O_2_ [87]. In the Fenton reaction, iron reacts with H_2_O_2_, and this reaction leads to the production of •OH. And it is a strong oxidizing agent, and it starts LPO [88]. The generation of ROS that depends on iron through the Fenton reaction leads to the process of ferroptosis [89]. Also, one of the features of ferroptosis is the accumulation of lipid peroxides, and they come from the oxidative damage of polyunsaturated fatty acids (PUFAs) in cellular membranes [90]. ROS, including •OH made by the Fenton reaction, attack and oxidize PUFAs, and this leads to the formation of lipid hydroperoxides [91]. The accumulation of lipid hydroperoxides breaks membrane integrity, harms cellular functions, and finally causes ferroptotic cell death [92]. Finally, the GSH system, which includes reduced GSH and enzymes that take part in its metabolism, is a very important regulator of ferroptosis [93]. GPX4 is an enzyme that plays a central role in protecting cells against ferroptosis [94]. GPX4 utilizes GSH as a cofactor to reduce lipid hydroperoxides and prevent their accumulation in cellular membranes [95]. By stopping the harmful effects of lipid peroxides, GPX4 keeps membrane integrity and protects cells from ferroptotic cell death [96]. Stopping the function or using up GPX4 leads to lipid peroxide detoxification not working well, cells being more likely to be affected by ferroptosis, and more ferroptotic cell death [97].

### 3.2 Cuproptosis

In contrast to iron-mediated redox cycling and ferroptotic pathways, copper-based systems introduce distinct catalytic and metabolic vulnerabilities. The following section therefore highlights copper ion-regulated phototherapeutic strategies and their unique mechanisms of action. Copper is a very important trace element in the human body, and it takes part in many important biological processes, including cellular respiration, antioxidant reactions, and the synthesis of neurotransmitters [98]. The balance of copper is very important for keeping the health of cells and organs [99]. But when copper ions build up too much, they may cause a series of toxic reactions, and finally cause cell damage and death [100].

#### 3.2.1 Molecular mechanisms of cuproptosis

**Fig. 4:**
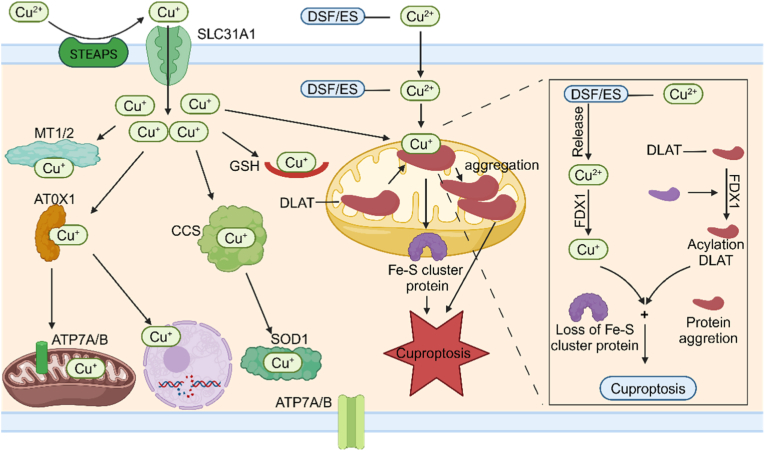
Cuproptosis is the way that too much copper ions build up in the cell. These ions attach to the acylated proteins in the mitochondrial TCA, causing the acylated proteins to clump together abnormally and the cell to lose ion sulfur cluster proteins. This then causes stress from damaged proteins, and finally leads to cell death. Created with BioRender.com.

Cuproptosis is a new kind of cell death caused by copper ions, with a complicated and different mechanism [101]. Regarding copper ion uptake and transport (Fig. 4), copper ions Cu^2+^ mainly enter cells through copper transporters on the cell membrane, and CTR1 is the main protein that takes in copper ions [[102], [103], [104]]. ATP7A and ATP7B are mainly in charge of controlling the process of getting rid of copper ions and where copper ions go inside the cell, so as to keep the amount of copper ions within the normal range for the body [48,105]. After entering the cell, copper ions usually attach to proteins that help copper ions like ATOX1 and COX17, and these proteins carry them to target enzymes or other important proteins [106]. Oxidative stress caused by copper ions also has an important part in copper death [107]. Cu^2+^ can help produce ROS through the Fenton and Haber-Weiss reactions [108]. These very reactive free radicals can damage lipids, proteins, and DNA inside the cell, and this causes oxidative damage. It also speeds up the cell death process because the cell is badly damaged [109,110]. Mitochondrial function that doesn't work well is another important sign of copper death [111]. Copper ions attaching to key enzymes inside the mitochondria disturbs the metabolic steps of the TCA. This makes ATP synthesis not work well and the cell not have enough energy, so the cell can't keep its normal physiological functions [112,113]. The loss of mitochondrial membrane potential is an important sign of cell death [114]. The loss of membrane potential means the balance of ions inside and outside the mitochondria is broken. This affects the stable state inside the cell and speeds up the cell death process [115]. Higher how easily things can pass through the inner membrane, stronger oxidative stress processes inside the mitochondria, and higher amounts of calcium ions inside the cell are all important things that happen during copper death [116].

### 3.3 Calcium overload

Calcium ions (Ca^2+^) are a type of messenger that's found everywhere inside cells, and they're called “second messengers”. Unlike classical metal ion-dependent regulated cell death pathways, calcium overload does not constitute an independent cell death program but instead functions as a potent stress amplifying mechanism that sensitizes tumor cells to phototherapy induced apoptosis or necrosis [117]. The endoplasmic reticulum is the main place where cells store calcium. It keeps high amounts of calcium inside itself by using a type of protein called sarco/endoplasmic reticulum Ca^2+^-ATPases, and these proteins help move calcium into the endoplasmic reticulum [118]. Mitochondria take up Ca^2+^ through the voltage dependent anion channel and the mitochondrial calcium uniporter, forming an ER-mitochondrial calcium signaling coupling [119]. This coupling depends on the structural integrity of mitochondria associated ER membranes, where the complex formed by inositol triphosphate receptor, glucose regulated protein 75, and VDAC1 serves as the core channel for calcium transport [120].

#### 3.3.1 Mechanisms of calcium overload

**Fig. 5:**
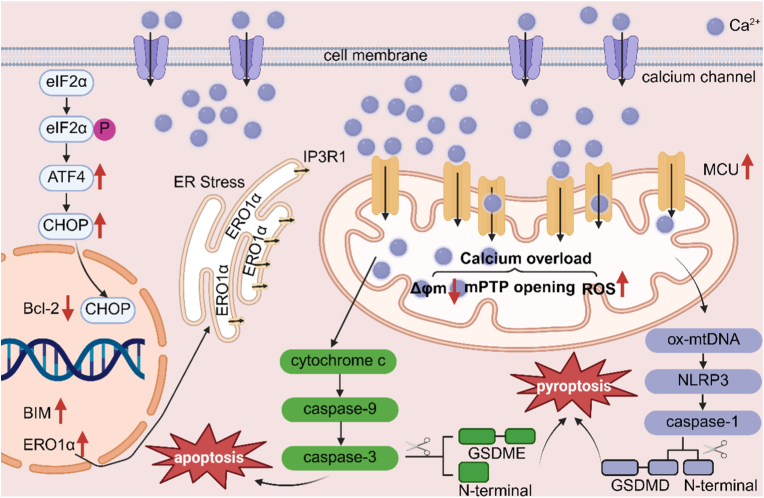
Mechanism of calcium overload. It will lead to the opening of transparent metastatic holes in mitochondria, the release of cytochrome C, the cascade activation of cytochrome C and the start of apoptosis process, which will trigger oxidative stress, cause a lot of praise, destroy cell structure and function, and eventually lead to death. Created with BioRender.com.

Calcium overload disrupts the mitochondrial membrane potential, leading to the opening of the mitochondrial permeability transition pore (MPTP) [121]. When the MPTP opens, a protein called cytochrome *c* is released from the mitochondria. This protein then starts a chain of reactions involving caspases, and these are proteins that help cells die. In the end, this whole process sets off apoptosis, which is the cell's planned, regular way of dying [122,123]. At the same time, excessive Ca^2+^ also stimulate the generation of ROS, causing oxidative stress, which further weakens mitochondrial function and forms a vicious cycle [124]. Meanwhile, the endoplasmic reticulum stores a large amount of Ca^2+^ [125]. When calcium overload occurs, the endoplasmic reticulum releases Ca^2+^ into the cytoplasm, which may be mediated by IP3 receptors or ryanodine receptors [126,127]. This excessive release brings about both endoplasmic reticulum stress and calcium signaling disorder, further exacerbating organelle dysfunction, disrupting the dynamic balance of intracellular Ca^2+^, and leading to more pathological reactions (Fig. 5) [128,129]. Moreover, cells usually rely on calcium pumps to actively expel Ca^2+^ to maintain homeostasis [130]. However, in the strategy of calcium overload, the dynamic balance of Ca^2+^ is often disrupted by inhibiting calcium extrusion or enhancing extracellular calcium influx [131,132]. Finally, calcium overload can also activate calcium dependent proteases and phospholipases, leading to the degradation of cytoskeletal proteins and the disruption of cell membrane integrity, ultimately triggering necroptosis or pyroptosis [133,134]. When the cell's cytoskeleton is destroyed, the cell loses its support and normal shape. At the same time, damage to the cell membrane makes the stuff inside the cell leak out [135,136]. Both are direct signs that the cell is dying. Therefore, calcium overload is discussed in this review as a noncanonical, metal associated synergistic mechanism rather than a defined MIDCD subtype, emphasizing its role in lowering cellular tolerance thresholds under photothermal or photodynamic activation.

### 3.4 Other metal ion-dependent cell death

Mg, Mn, and Zn may also lead to cell death through mechanisms such as causing oxidative stress, mitochondrial dysfunction, and apoptosis (Table 1) [177,178]. But they are different in specific goals and specific mechanisms [179]. It should be noted that the concentration of ions within cells is usually accurate [180]. Ions are usually necessary for normal cell physiological functions at low concentrations, and only at abnormal concentrations can they cause cell death [181].

**Table 1:** Mg, Mn, and Zn cause cell death by damaging mitochondria, changing cell membrane permeability, disturbing ion balance inside cells, and blocking DNA synthesis and repair. They also activate processes like oxidative stress, endoplasmic reticulum stress, autophagy, and zinc induced cell death, which finally lead to apoptosis or necrosis.

Ion	Combined therapy	Imaging	Tumor model	Administration	Mechanism	Ref
**Mg**	PTT, PDT, CDT, chemotherapy, immunotherapy	MRI/NMR/PAI/BLI	143b, 4T1, CT26, LLC, U87, MCF-7, HeLa	intranasal or intratumoral or intraperitoneal injection	affecting cell membrane stability and interfering with cellular metabolism;	[[137], [138], [139], [140], [141], [142], [143], [144], [145], [146], [147], [148], [149]]
**Mn**	PTT, PDT, CDT, SDT, chemotherapy, immunotherapy	MRI/NMR/PAI/BLI	4T1, CT26, MCF-7, HGP2, B16F10, H22, U87	intranasal or intratumoral or intraperitoneal injection	generating oxidative stress, disrupting intracellular ion balance and affecting the activity of intracellular enzymes;	[[150], [151], [152], [153], [154], [155], [156], [157], [158], [159], [160], [161], [162], [163]]
**Zn**	PTT, PDT, CDT, SDT, chemotherapy, immunotherapy	MRI/NMR/PAI/BLI	CT26, 4T1, B16, A549, KP, H22	intranasal or intratumoral or intraperitoneal injection	lysosomal zinc dependent cell death, disrupting intracellular zinc homeostasis, and affecting intracellular signal transduction;	[[164], [165], [166], [167], [168], [169], [170], [171], [172], [173], [174], [175], [176]]

## 4 Synergistic effects of ferroptosis and phototherapy

It should be emphasized that not all iron ion mediated oxidative damage can be equated with ferroptosis, as bona fide ferroptotic cell death strictly requires iron dependent LPO accompanied by disruption of the GSH/GPX4 axis. Building on this mechanistic clarity, recent advances have increasingly shifted toward multifunctional nanoplatforms that integrate phototherapy, catalytic therapy, and diagnostic capabilities, thereby enabling more precise and efficient therapeutic interventions. Within such integrated designs, the combination of ferroptosis with PDT or PTT emerges as a particularly robust synergistic strategy, since phototherapy generated ROS and localized hyperthermia accelerate LPO and thereby reinforce ferroptotic execution. Conversely, ferroptosis associated metabolic reprogramming facilitates the conversion of intracellular H_2_O_2_ into O_2_, partially alleviating tumor hypoxia and in turn improving the efficacy of oxygen dependent PDT. Simultaneously, photothermal heating enhances Fenton-type reactions and promotes cellular uptake of therapeutic agents, further amplifying ROS production. Through this bidirectional reinforcement, a self-propagating cycle of oxidative stress and regulated cell death is established, allowing effective tumor ablation at reduced drug dosages and lower light intensities. Collectively, the synergistic coupling of ferroptosis with phototherapy not only strengthens antitumor efficacy but also exemplifies a promising paradigm for precision cancer therapy, providing a conceptual foundation for the following section, which focuses on how integrated nanoplatform designs further refine therapeutic accuracy and outcomes.

### 4.1 Mechanisms of ferroptosis amplified phototherapy

Rather than acting as an independent cytotoxic mechanism, phototherapy primarily functions as a stress amplifier in ferroptosis based synergistic systems. By rapidly elevating intracellular oxidative pressure, phototherapy accelerates iron driven LPO and pushes cells beyond the buffering capacity of ferroptosis defense pathways, such as GSH dependent antioxidant systems. Importantly, ferroptosis redefines the biological outcome of phototherapy by converting transient oxidative stress into irreversible membrane damage. This functional coupling, rather than redundant ROS generation, underlies the observed “1 + 1 > 2” synergistic effect.

#### 4.1.1 Ferroptosis amplified PDT

**Fig. 6:**
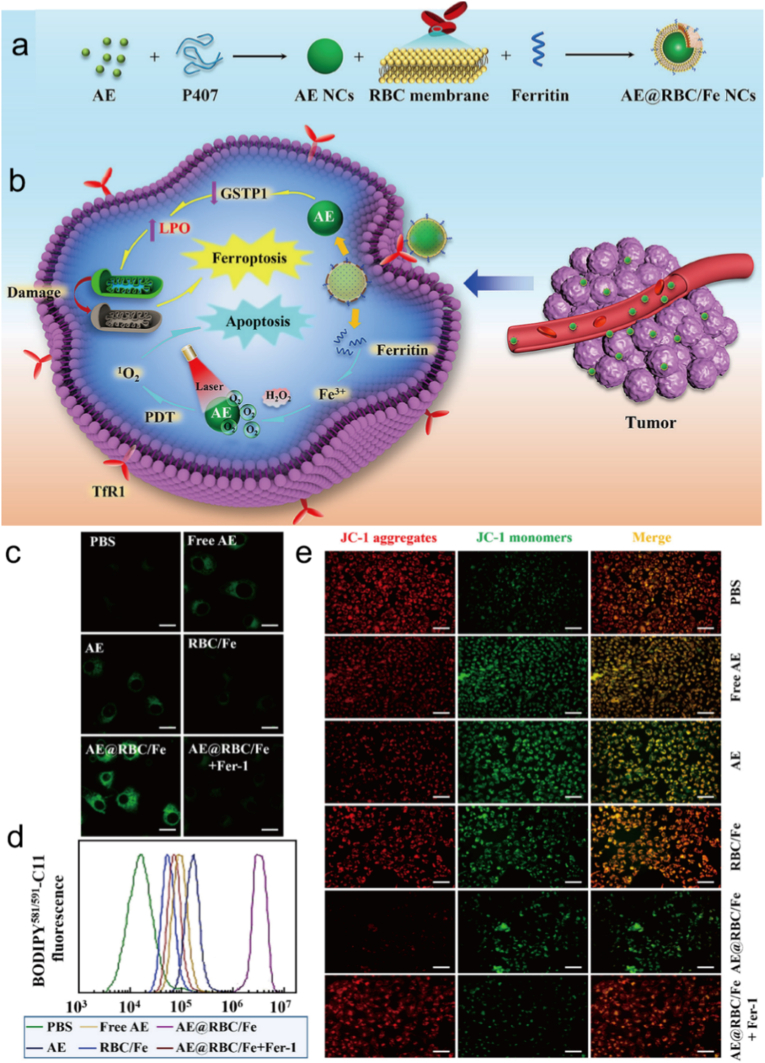
(a) Preparation procedure of AE@RBC/Fe NCs. (b) Schematic illustration of PDT/ferroptosis synergistic therapy after intravenous injection of AE@RBC/Fe NCs. (c) CLSM images (Scale bar: 10 μm) of cellular LPO by BODIPY581/591-C11 staining after the treatment with different formulations. (d) Flow cytometry assay of cellular LPO by BODIPY581/591-C11 staining after the treatment with different formulations. (e) Mitochondria membrane potential analysis by JC-1 staining after the treatment with different formulations (Scale bar: 50 μm). Reproduced with permission [195]. Copyright 2022, ELSEVIER.

Ferroptosis can enhance PDT in multiple ways. One way is by generating more cytotoxic free radicals. PDT relies on cytotoxic ROS produced from molecular oxygen absorbing energy under irradiation to kill tumor cells [182,183]. Ferroptosis is an iron dependent form of programmed cell death [184]. During this process, iron can react with excess hydrogen peroxide in tumors through the Fenton reaction (${Fe}^{2+}+H_{2}O_{2}\;\to {Fe}^{3+}+{\left(OH\right)}^{-}+\cdot OH$) to generate **·**OH [185,186]. Hydroxyl radicals are also a type of ROS that can further oxidize PUFAs to produce LPO, which damage cell structure and integrity [187,188]. This supplements the types and quantities of ROS produced by PDT, enhancing oxidative stress damage to tumor cells [189]. In addition, it can also enhance oxidative stress damage [190]. The ROS produced by PDT, along with the hydroxyl radicals and lipid peroxides generated by ferroptosis, act on tumor cells together [191]. This significantly increases the intracellular oxidative stress level beyond the cell's own antioxidant capacity, causing more severe damage to cell structure and function, and thus improving the therapeutic effect [192]. For example, the accumulation of lipid peroxides can destroy the integrity and stability of the cell membrane, affect the normal physiological functions of the cell, and ultimately promote the death of tumor cells [193,194]. Wu et al., first clarified the metabolic pathway of AE-induced ferroptosis, and then developed a novel ferritin modified biomimetic AE nanocrystals (AE@RBC/Fe NCs) by coextruding AE nanocrystals, prefabricated red blood cells membranes and Fe for the synergistic treatment of PDT and ferroptosis (Fig. 6a–b) [195]. Upon uptake by tumor cells, over expressed phospholipases D in the tumor cell could disintegrate the phospholipid component of the outer layer of AE@RBC/Fe NCs, resulting in the exposure and release of the AE NCs inner core (Fig. 6c–e). AE not only exerts the properties of a photosensitizer to convert intracellular oxygen to ^1^O_2_ under laser irradiation, but also initiates ferroptosis by inhibiting the activity of GSH S-transferase P1 (GSTP1). More importantly, thanks to the involvement of ferritin, both AE-mediated ferroptosis and PDT were effectively enhanced due to Fe^3+^ supply and oxygen replenishment.

#### 4.1.2 Ferroptosis amplified PTT

**Fig. 7:**
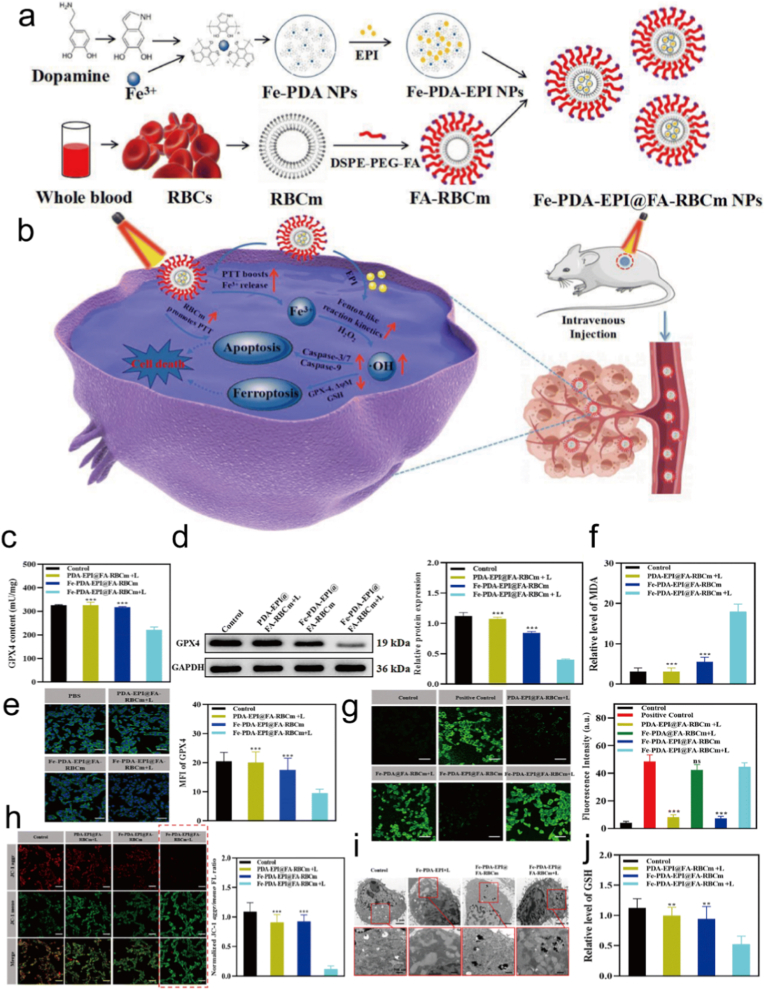
(a) Preparation of Fe-PDA-EPI@FA-RBCm NPs. (b) A schematic illustration of NIR-mediated PTT and Fenton-like mechanisms that induce synergistic Ferroptosis-PTT. (c) GPX4 activity of 4T1 cells following 24 h of incubation with PDA-EPI@FA-RBCm NPs + L, Fe-PDA-EPI@FA-RBCm, and Fe-PDA-EPI@FA-RBCm NPs + L. (d-e) The expression levels of GPX4 protein in 4T1 cells after different treatments were detected by western blot analysis and immunofluorescence. (f) Based on the MDA content of different groups of 4T1 cells, the Fe-PDA-EPI@FA-RBCm NPs + L group produced the highest levels of MDA. (g) CLSM images and fluorescence semi-quantitative statistics of 4T1 cells incubated with drug-loaded NPs and treated with DCFH-DA. (h) CLSM pictures and fluorescence semi-quantitative statistics of 4T1 cells stained with JC-1 after treatment with drug-loaded NPs. (i) Effects of different treatments on mitochondrial morphology in 4T1 cells. (j) Intracellular total GSH content of 4T1 cells following 24 h of incubation with different treatments. Scale bar represents 50 μm. Reproduced with permission [204]. Copyright 2023, Royal Society of Chemistry.

The mechanism of combined PTT and ferroptosis therapy is mainly reflected in two aspects. First, PTT can enhance ferroptosis [196]. PTA convert light energy into heat energy under NIR irradiation [197]. The local temperature increase can promote the release of more Fe from compounds or carriers, providing the necessary source for ferroptosis and thereby enhancing the ferroptosis effect [198]. It can also accelerate LPO, intensify the ferroptosis process, and simultaneously disrupt the antioxidant system of tumor cells, reducing the intracellular GSH levels, decreasing the antioxidant capacity of cells, disrupting the redox balance, and enhancing the induction of ferroptosis [199]. Second, ferroptosis can also optimize PTT [200]. The process of ferroptosis can reduce the expression of HSPs in PTT, weaken the tolerance to PTT, and enhance the efficacy of PTT [201,202]. Moreover, the membrane damage and increased permeability caused by ferroptosis facilitate the better entry of PTA into the cell interior, improving the effectiveness of PTT and making tumor cells more susceptible to thermal damage and death [203]. For example, Yu et al., proved that Fe-PDA-EPI@FA-RBCm NPs were successfully constructed to synergistically deliver EPI, Fe^3+^ and PDA for low temperature PTT amplified ferroptosis (Fig. 7a–b) [204]. The built in photothermal effect and intracellular acidic pH jointly promoted Fenton-like reaction kinetics, which compensated for the low efficiency of ferroptosis alone, inducing highly efficient ferroptosis-PTT in vitro. Indeed, GPX4 expression levels of the ferroptosis marker were significantly down regulated during ferroptosis-PTT treatment in vitro and in vivo (Fig. 7c–j). Furthermore, assisted by mild PTT, the in vivo results revealed pronounced tumor growth suppression, suggesting that photothermal activation cooperatively amplifies ferroptosis and apoptosis pathways to enhance therapeutic outcomes.

### 4.2 Fe-based components of combination treatments

Iron based nanocarriers, including iron organic frameworks and hemoglobin nanoparticles, effectively deliver PSs and release Fe^2+^/Fe^3+^ within the TME [205,206]. These ions catalyze the Fenton reaction, generating ROS that enhance LPO and induce ferroptosis [207,208].

#### 4.2.1 Fe_3_O_4_-based nanomaterials

Fe_3_O_4_-based nanomaterials have emerged as versatile agents to potentiate PTT and PDT owing to their unique catalytic, photothermal, and immunomodulatory properties [209]. In the acidic TME, Fe_3_O_4_ releases Fe^2+^/Fe^3+^ to trigger Fenton or Fenton-like reactions, thereby generating hydroxyl radicals and amplifying ROS for enhanced PDT or CDT, while also inducing ferroptosis through LPO [210]. Moreover, their integration with photosensitizers or photothermal agents improves NIR absorption, photothermal conversion, and oxygen generation to overcome tumor hypoxia, further augmenting PDT efficacy [211]. For instance, Qin et al. constructed Fe_3_O_4_/ICG-lactate oxidase/catalase coloaded hybrid nanogels, which regulate O_2_ redistribution and H_2_O_2_ activation to enhance both CDT and PDT [212]. Liang et al. designed ultrasmall Fe_3_O_4_@PGL NPs that enable imaging guided PDT while released Fe ions induce ferroptosis through the Fenton reaction [20]. Ding et al. developed a dual responsive hydrogel containing PpIX-modified Fe_3_O_4_ nanoparticles and anti-PD-L1 prodrug, where combined PDT and CDT generated amplified ROS to induce ICD and checkpoint blockade for synergistic immunotherapy [24]. Similarly, Chin et al. fabricated Fe_3_O_4_@chlorophyll clustered nanoparticles that combined PDT-induced singlet oxygen with CDT-mediated ferroptosis, while reprogramming the immunosuppressive TME [213]. Xu et al. synthesized yolk-shell Fe_3_O_4_@Carbon@Pt-Ce6 nanozymes with MRI capacity and high photothermal conversion efficiency for catalytic-PDT-PTT therapy [214]. Zhou et al. developed Fe_3_O_4_@Au nanocomposites with strong photothermal conversion efficiency and singlet oxygen generation under a single 808 nm laser, enabling efficient magnetic targeting assisted PTT/PDT [215]. Furthermore, Zhang et al. demonstrated Fe_3_O_4_@BSA-Ce6 nanoparticles that simultaneously induce apoptosis via PDT and ferroptosis via LPO, achieving synergistic tumor eradication [216]. Collectively, these studies highlight the multifaceted role of Fe_3_O_4_ in modulating the TME, enhancing ROS production, and integrating PTT, PDT, CDT, and ferroptosis for highly efficient and synergistic cancer therapy.

#### 4.2.2 Fe_2_O_3_-based nanomaterials

Nowadys, Fe_2_O_3_ has been extensively exploited to construct multimodal nanoplatforms that synergistically amplify PDT and PTT, owing to its tailorable morphology, excellent Fenton-like catalytic activity and appreciable NIR absorption [217]. Zhao et al. designed FeTCPP/Fe_2_O_3_ MOF “nanorice” through a liquid diffusion strategy. The Fe nodes efficiently catalyze endogenous H_2_O_2_ to produce **·**OH and simultaneously generate O_2_, relieving tumor hypoxia and markedly promoting singlet oxygen generation of the porphyrin photosensitizer, thus achieving high level PDT/CDT cooperation [218]. Curcio et al. further prepared IONF@CuS hybrids with a γ-Fe_2_O_3_ nanoflower core and a spiky CuS shell the nanoplatform exhibits a photothermal conversion efficiency of 42% under 808 nm irradiation and a specific absorption rate of ∼350 W/g under alternating magnetic field, enabling concurrent PTT, magnetic hyperthermia and PDT and leading to complete tumor regression in a single treatment [219]. To maximize the oxygen supply and ROS-cascade capacity of Fe_2_O_3_, Gan et al. constructed an MgO_2_-Fe_2_O_3_/CNx-Ce6 nanoreactor that integrates MgO_2_-mediated H_2_O_2_ self-supply with the dual enzyme mimetic activities (CAT/POD) of Fe_2_O_3_. Under 660 nm light for 4 min the platform continuously converts intertumoral H_2_O_2_ into O_2_ and **·**OH, yields abundant ROS for PDT/CDT, and reduces the survival rate of breast cancer cells to 14% while suppressing tumor angiogenesis in vivo [147]. Additionally, the superparamagnetic of Fe_2_O_3_ endows nanocarriers with magnetically targeted delivery and MRI visibility. Haimov-Talmoud et al. covalently conjugated mTHPC to Ce-doped γ-Fe_2_O_3_ nanoparticles, and the application of an external magnetic field doubled drug accumulation in tumors. Subsequent PDT produced significant tumor shrinkage in mice [220]. Collectively, Fe_2_O_3_ not only acts as an intrinsic photothermal agent for direct heat generation but also amplifies oxidative stress via Fenton chemistry, alleviates hypoxia, and integrates magnetic targeting, imaging and drug delivery functionalities, offering a versatile and powerful nano engineering strategy for “visualizable targeted multi modal” cancer phototherapy.

#### 4.2.3 Fe^3+^-based complexes

**Fig. 8:**
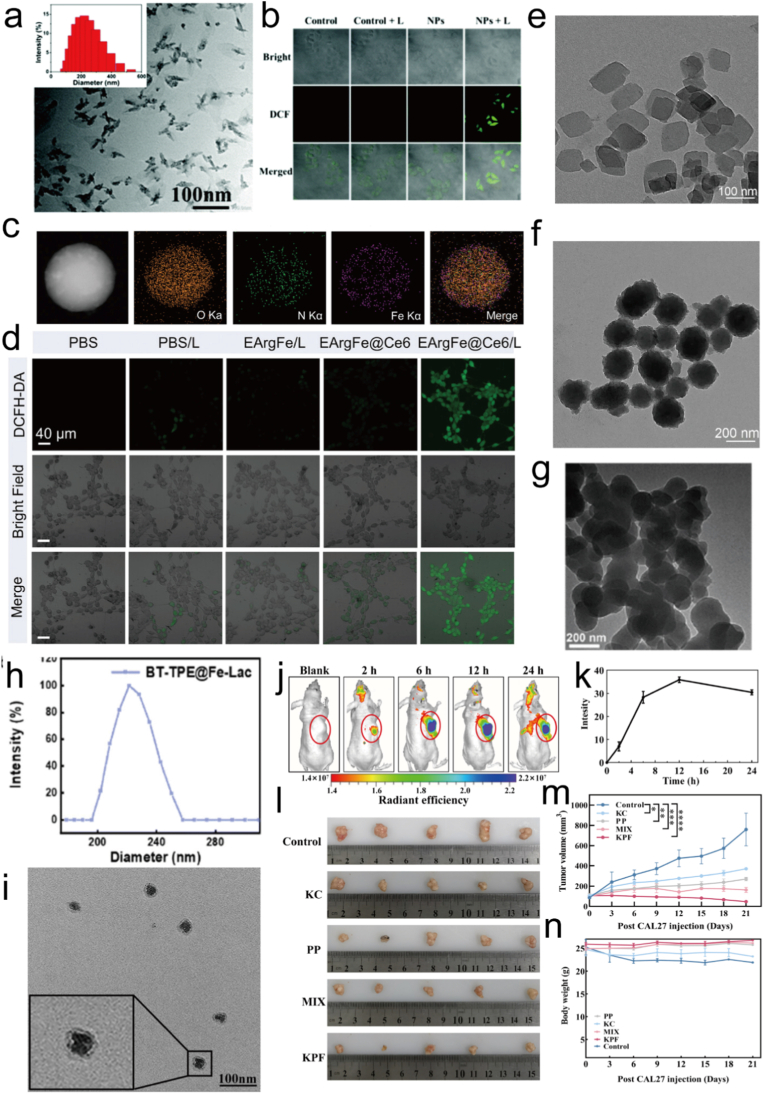
(a) TEM image of the Fe-IBDP NPs, with a DLS profile shown in the inset. (b) Generation of intracellular ROS mediated by Fe-IBDP NPs (concentration of IBDP, 0.5 μg mL^−1^) with 540 nm LED irradiation at 10 mW cm^−2^ for 20 min as indicated by the fluorescence of DCF. Scale bars, 20 μm. Reproduced with permission [225]. Copyright 2019, Royal Society of Chemistry. (c) Elemental mapping of O, N, and Fe in EArgFe. (d) CLSM observation of intracellular ROS levels after different treatments. DCFH-DA was chosen as the ROS probe. Reproduced with permission [226]. Copyright 2023, Wiley. (e) TEM images of PCN. (f) TEM images of LPC@PCN@PDA. Reproduced with permission [227]. Copyright 2024, Springer Nature. (g) TEM image of BT-TPE@Fe-Lac. (h) DLS of BT-TPE@Fe-Lac. Reproduced with permission [228]. Copyright 2025, Royal Society of Chemistry. (i) TEM image of KPF NPs. (j) Real-time imaging on nude mice bearing CAL-27 tumors after the administration of KPF NPs (200 μL) at a dosage of 5 mg/kg corresponding to body weight. The tumor was identified in the region labeled by the yellow circle. (k) Quantitative analysis of hybrid KPF NPs in tumor tissue at different time points. (l) Representative images of tumors in the CAL-27 xenografts models with indicated treatments. (m) Change in the volume of the tumor after administration of indicated treatments (5 mg/kg based on PP, 200 μL) under irradiation (100 mW/cm^2^, 5 min). The significance of the statistical level is ∗ p < 0.05, ∗∗p < 0.01, ∗∗∗p < 0.001, ∗∗∗∗p < 0.0001. (n) Body weight change analysis of tumor-bearing mice. Reproduced with permission [229]. Copyright 2024, MDPI. (For interpretation of the references to colour in this figure legend, the reader is referred to the Web version of this article.)

Fe^3+^-based coordination complexes have been extensively investigated as multifunctional nanoplatforms to enhance PTT and PDT owing to their unique redox activity and TME responsiveness [221]. Specifically, Fe^3+^ participate in Fenton or Fenton-like reactions, where the Fe^3+^/Fe^2+^ redox cycle catalyzes H_2_O_2_ to generate highly cytotoxic **·**OH, thereby amplifying the ROS mediated tumor killing effect of PDT [222,223]. In addition, Fe^3+^ can decompose endogenous H_2_O_2_ to O_2_, alleviating tumor hypoxia and improving the photosensitizer efficiency during PDT, while its coordination with polyphenols or porphyrins can further enhance light absorption and photothermal conversion for PTT [224]. Song et al. constructed Fe-IBDP coordination polymer nanoparticles, in which Fe^3+^ initially quenched the photosensitizer but subsequently released BODIPY derivatives under TME activation to produce singlet oxygen, achieving precise PDT (Fig. 8a–b) [225]. Shi et al. designed EArgFe@Ce6 nanoplatforms by coordinating EGCG with Fe^3+^, which provided efficient mild PTT and relieved hypoxia to enhance PDT while simultaneously triggering NO gas therapy under single 660 nm irradiation (Fig. 8c–d) [226]. Similarly, Liu et al. reported LPC@PCN@PDA/Fe^3+^-AS1411 nanoplatforms, where Fe^3+^ catalyzed H_2_O_2_ to relieve hypoxia and promoted CDT, thus synergistically enhancing PDT and chemotherapy (Fig. 8e–f) [227]. Feng et al. synthesized a glycosylated Fe^3+^ photosensitizer (BT-TPE@Fe-Lac) that combined PDT and CDT through TME responsive Fe^3+^ coordination, producing both singlet oxygen and hydroxyl radicals to synergistically inhibit tumor growth (Fig. 8g–h) [228]. Moreover, Li et al. constructed protoporphyrin IX/Fe^3+^ hybrid nanoparticles with HIF-1α inhibitor, where Fe^3+^ promoted ROS production via Fenton reaction and acted synergistically with HIF-1α inhibition to enhance PDT efficacy (Fig. 8i–n) [229]. Collectively, these studies highlight that Fe^3+^ complexes can simultaneously modulate hypoxia, amplify ROS generation, and integrate multiple therapeutic modalities, making them powerful candidates for advanced PTT/PDT synergistic cancer therapy.

### 4.3 Ferroptosis inducers

In addition to the enhancing the Fe by increasing the iron ions content of cancer cells, there are also reports suggest that ROS in cancer cells can also be amplified via alternative pathways in Table 2. The strategic combination of ferroptosis inducers, buthionine sulfoximine (BSO), sorafenib, erastin and RSL3 with advanced nanoplatforms demonstrate significant potential in cancer therapy [257,258]. These inducers function by elevating intracellular iron, catalyzing Fenton reactions to generate ROS, leading to lethal LPO accumulation and ferroptosis initiation.

**Table 2:** Summary of other ferroptosis inducers for combination cancer therapy.

Ferroptosis inducer	Mechanism	Core Material	Tumor	Ref
BSO	inhibition of glutamylcystei-ne synthetase	Er@NaYF4@mSiO_2_@lipsome-Ce6-BSO	B16/F10	[230]
		BSO-MOF-HA	4T1	[231]
Sorafenib	inhibiting system Xc-	MnO_2-_SOR-Ce6@PDA-PEG-FA	SMMC-7721	[232]
		5-ALA-derived PpIX	SiHa	[233]
		AQ4N-Ir1-sorafenib-liposome	HepG2	[234]
		SCPP	4T1	[235]
		CM-HSA^DSP^@[PS-Sor]	4T1	[236]
		Ce6@SRF@RDV	MG63	[237]
		hPPAA18C6@Ce6	B16F10	[238]
		SRF@CuSO_4.5_H_2_O@IR780	K7M2	[239]
		SRF@Hb-Ce6	4T1	[240]
		Sor-Ce6	CAL-27	[241]
		Ce6-Sor@PFC-F127	4T1	[242]
		SRF@FeIIITA–NAPP	HeLa	[243]
		MIL-53@cMBP@ST/Ce6	CAL-27	[244]
		BCFe@SRF	NIH 3T3	[245]
Erastin	inhibiting system Xc-	Er/RB@ExosCD47	Hepa1-6	[246]
		ZCND	4T1	[247]
		Ce6-erastin	CAL-27	[248]
		PPa@Era NAs	4T1	[249]
		FECTPN	MCF-7	[250]
		FIN + IKE + Ce6	LLC	[251]
		DMONs-HE@BSA	U87	[252]
RSL3	inhibition of the activity of GPX4	CR-NML	4T1	[253]
		HAFeR	MB49	[254]
		PLA@R	LLC	[255]
		D-NP_VR_	4T1	[256]

**Fig. 9:**
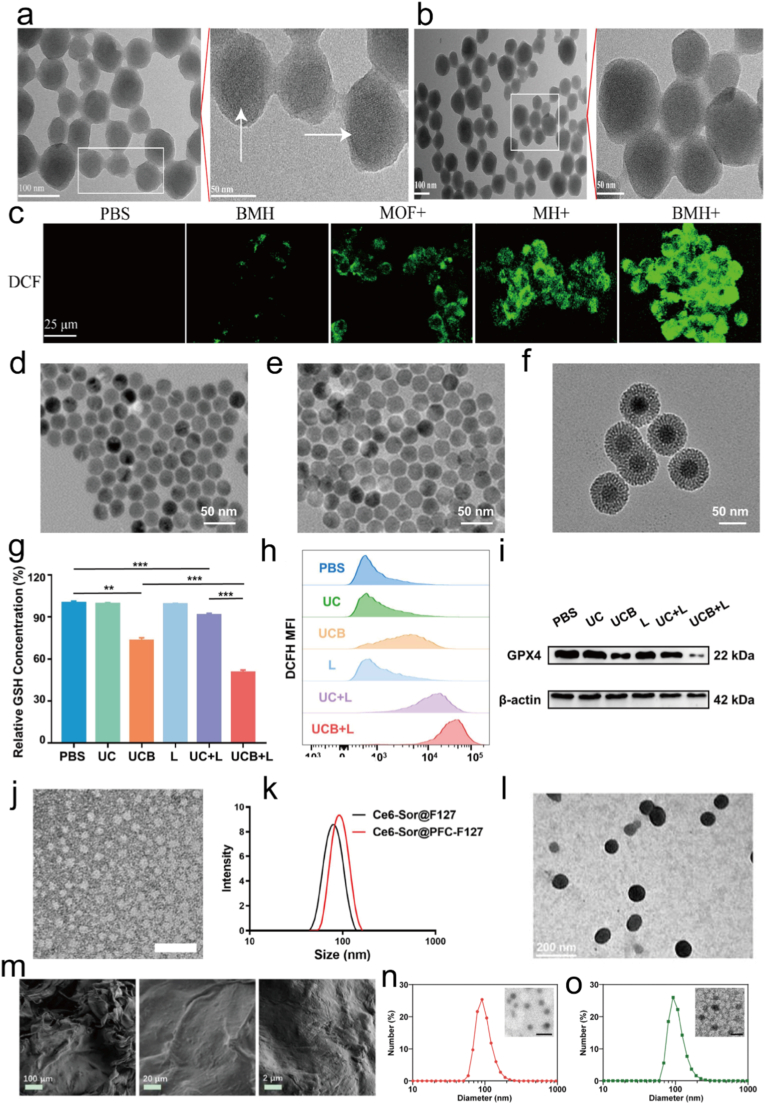
(a) The typical image of MOF detected by TEM. (b) The typical image of BMH detected by TEM. (c) CLSM detection of intracellular ROS production after treated MOFs with or without irradiation (scale bar: 25 μm). Reproduced with permission [259]. Copyright 2023, ELSEVIER. (d) TEM image of NaYF4:Yb,Er. (e) TEM image of UCNPs. (f) TEM image of UCNP@mSiO_2_ NPs. (g) Cell viabilities of B16/F10 cells incubated with different concentration of UCB for 6 h, 12 h and 24 h. (h) Cell viabilities of B16/F10 cells in response to different formulations after 12 h of incubation with or without laser irradiation (980 nm, 0.7 W/cm^2^, 10 min). (i) Apoptosis illustration of B16/F10 cells treated with PBS, UC and UCB for 12 h in the presence or absence of irradiation by flow cytometry. Reproduced with permission [260]. Copyright 2021, ELSEVIER. (j) TEM image of Ce6-Sor@PFC-F127 micelles (scale bar: 100 nm). (k) Dynamic light scattering measurement of micelles formed by pristine F127 and PFC-F127. Reproduced with permission [242]. Copyright 2024, American Chemical Society. (l) Representative TEM image of Ce6-erastin. Reproduced with permission [261]. Copyright 2019, Lvyspring International Publisher. (m) SEM images of FMPEG. Reproduced with permission [262]. Copyright 2024, ELSEVIER. (n) DLS measurement of NPVR and D-NPVR. (o) TEM image of NPVR and D-NPVR. Reproduced with permission [256]. Copyright 2023, Royal Society of Chemistry.

BSO, a γ-glutamyl-cysteine synthetase inhibitor that blocks de-novo GSH synthesis, has been integrated by Wang et al. into BSO-MOF-HA nanoparticles co-loaded with the photosensitizer TCPP. Upon irradiation the preexisting GSH pool is rapidly exhausted, 4T1 cells lose their “reductive shield” against LPO, and the combined ferroptosis-PDT response ignites dendritic cell maturation and T-cell infiltration, culminating in robust ICD (Fig. 9a–c) [259]. Li et al. further engineered upconversion nanoparticles NaYF_4_:Yb, Er@NaYF_4_@mSiO_2_@liposome co-encapsulating Ce6 and BSO. BSO-mediated GSH depletion prevents GPX4 from detoxifying PDT-generated •OH, lipid ROS accumulate to trigger ferroptosis, and apoptotic signaling is simultaneously amplified, leading to pronounced suppression of melanoma growth (Fig. 9d–i) [260]. Sorafenib, a multichines inhibitor that additionally blocks system Xc- and down regulates GPX4, was incorporated by Chen et al. into a ZCND-Erastin/PAA:F127 composite hydrogel. Under NIR exposure the carbon nano dodecahedron simultaneously delivers photothermal heat and ROS, while sorafenib silences both HSP70 and GPX4, dismantling tumor intrinsic antioxidant and antishock defenses and achieving zero recurrence in a post-surgical recurrence model [247]. Ren et al. coloaded sorafenib and Ce6 into oxygen self-supplying PFC-F127 micelles. Sorafenib-mediated GPX4 inhibition coupled with perfluorocarbon enhanced oxygenation alleviates tumor hypoxia, amplifies lipid ROS and ^1^O_2_ accumulation, and breaks the hypoxia imposed resistance of breast cancer to PDT (Fig. 9j–k) [242]. Erastin, a system Xc-inhibitor that curtails cystine uptake and GSH production, was employed by Zhu et al. to fabricate Ce6-erastin supramolecular nanodrugs via hydrogen-bond/π-π stacking. Erastin-mediated SLC7A11 suppression disables cellular ROS scavenging, PDT-induced oxygen consumption is counter balanced by Fenton chemistry, and the reinforced LPO wave significantly potentiates oral tongue squamous cell carcinoma phototherapy (Fig. 9l) [261]. Xu et al. constructed the FMPEG hydrogel system incorporating Fe/Mn-polydopamine nanoparticles and the ferroptosis inducer piperazine erastin, which amplified ROS generation and downregulated GPX4 and xCT expression upon NIR laser irradiation, thereby achieving synergistic antitumor effects of PTT and CDT with enhanced immune activation for breast cancer ablation and metastasis prevention (Fig. 9m) [262]. RSL3, a covalent GPX4 inhibitor that aborts the reduction of lipid hydroperoxides, was co-encapsulated by Zhang et al. inside a singlet oxygen cleavable D-NPVR nanocarrier with verteporfin. Light triggered ^1^O_2_ simultaneously oxidises GSH and disassembles the nanoparticle, liberated RSL3 completely inactivates GPX4, and the resulting “PDT apoptosis plus GPX4-ablated ferroptosis” cascade elicits potent tumor regression in 4T1 bearing mice (Fig. 9n–o) [256]. Collectively, BSO cuts off the GSH source, sorafenib and erastin block the xCT import route and down regulate GPX4, and RSL3 executes GPX4. When these inducers meet the ROS or hyperthermia generated by PTT/PDT, the cellular antioxidant system collapses, lipid peroxides accumulate to lethal levels, and immunogenic danger signals are emitted, forging a tridirectionally positive feedback loop among phototherapy-ferroptosis-immunity and offering a clear mechanistic blueprint and versatile nanoplatform paradigms for next generation photo controllable ferroptosis sensitized cancer therapy.

## 5 Synergistic effects of cuproptosis and phototherapy

The synergy between cuproptosis and phototherapy arises from reinforced proteotoxic stress and mitochondrial metabolic collapse. Phototherapy induced oxidative stress or hyperthermia accelerates copper redox cycling and intracellular copper accumulation, thereby enhancing copper binding to lipoylated components of the TCA. This process promotes protein aggregation and destabilization of iron sulfur cluster containing enzymes, compromising mitochondrial respiration. The convergence of phototherapy triggered stress and copper induced proteotoxicity establishes a nonredundant cytotoxic pathway distinct from conventional oxidative damage, accounting for the enhanced therapeutic efficacy.

### 5.1 Mechanisms of cuproptosis amplified phototherapy

**Fig. 10:**
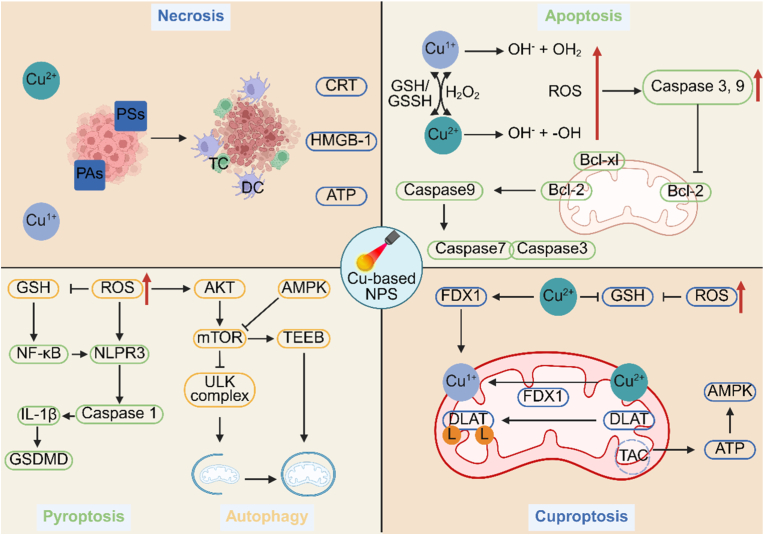
Schematic diagrams of Copper-based nanomaterials enhance PTT/PDT efficacy by inducing tumor cell death via photothermal effects, ROS elevation, apoptosis, pyroptosis, and autophagy modulation, offering new cancer treatment strategies. Created with BioRender.com.

As shown in Fig. 10, Copper-based nanomaterials induce tumor cell death by synergistically integrating PTT and PDT through multiple mechanisms [263,264]. They generate localized photothermal effects on tumor cell membranes, leading to rupture, ATP depletion, and necrosis [265]. Release Cu^+^ to elevate intracellular ROS levels, impair mitochondrial function, and activate apoptosis related proteins such as Bax and Caspase-3/9 [266]. Trigger pyroptosis under NIR irradiation by activating the NLRP3 inflammasome and downstream effectors including Caspase-1 and GSDMD and modulate intracellular Cu^+^/Cu^2+^ levels and signaling pathways such as mTOR and ULK1 to alter metabolism and energy homeostasis, inducing autophagy that can shift from a survival response to autophagic cell death [267,268]. Collectively, in contrast to ferroptosis, the synergy between cuproptosis and phototherapy is governed by the convergence of metabolic vulnerability and stress sensitization rather than generalized oxidative damage. Phototherapy induced stress facilitates intracellular copper accumulation and redox cycling, which selectively destabilizes lipoylated mitochondrial enzymes and iron sulfur cluster containing proteins. This copper specific proteotoxic stress fundamentally alters the cellular response to phototherapy, shifting it from oxidative injury toward metabolic collapse. Thus, the synergistic efficacy arises from pathway specific coupling rather than overlapping mitochondrial damage. And these interconnected pathways significantly also enhance the therapeutic efficacy of PTT/PDT and provide novel strategies for cancer treatment.

#### 5.1.1 Cuproptosis amplified PDT

**Fig. 11:**
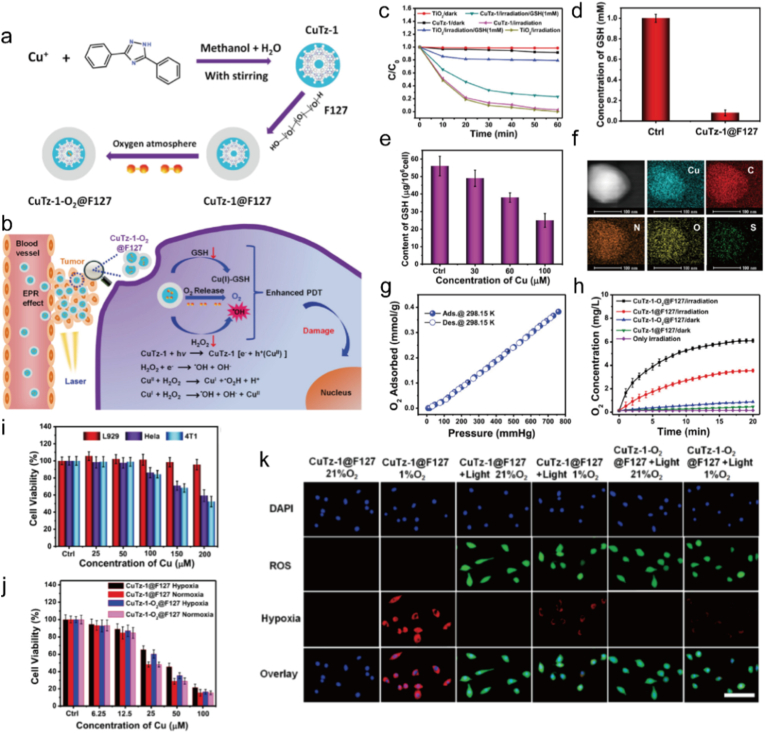
(a) Schematic illustration of CuTz-1-O_2_@F127 preparation and its application for amplified PDT. (b) Mechanism of amplified PDT using CuTz-1-O_2_@F127. (c) Degradation of RhB by CuTz-1@F127 compared to TiO_2_ in the presence or absence of GSH. (d) GSH concentration in supernatant after mixing with CuTz-1@F127. (e) Intracellular GSH depletion with increasing CuTz-1@F127 concentrations. (f) HAADF image and EDS mapping of a single CuTz-1@F127@GSH. (g) Oxygen adsorption-desorption isotherm of CuTz-1@F127 at 760 mmHg and 25 °C. (h) Oxygen production and release profile of CuTz-1-O_2_@F127. (i) In vitro cell viability of L929, HeLa, and 4T1 cells after 24 h incubation with CuTz-1-O2@F127. (j) In vitro PDT efficacy of 4T1 cells treated with CuTz-1@F127 and CuTz-1-O_2_@F127 under 808 nm irradiation in hypoxic or normoxic conditions after 24 h incubation. (k) ROS and hypoxia generation in cells incubated with CuTz-1@F127 or CuTz-1-O_2_@F127, with or without 808 nm laser irradiation. Reproduced with permission [272]. Copyright 2021, Elsevier.

In PDT, Cu^+^/Cu^2+^ continuously generate ROS via Fenton like reactions, causing LPO, protein denaturation, and DNA damage [269]. They also accumulate in mitochondria, leading to inactivation of tricarboxylic acid cycle (TCA) enzymes, mitochondrial membrane potential collapse, and cytochrome *c* release, thereby significantly enhancing tumor cell sensitivity to PDT-induced apoptosis [270]. The mechanisms include not only depletion of mitochondrial GSH, weakening the antioxidant system, but also disruption of mitochondrial nuclear signaling, inhibiting survival pathways like NF-κB [271]. Cai et al. developed a CuTz-1-O_2_@F127 MOF that acts as a photosensitizer to produce ROS, carries O_2_, and adsorbs GSH, achieving a triple synergy of “ROS burst + oxygen supply + GSH depletion” (Fig. 11a–b). This MOF exhibits high tumor selectivity, good biocompatibility, and metabolic clearance, showing significant potential for clinical application (Fig. 11c–k).

#### 5.1.2 Cuproptosis amplified PTT

**Fig. 12:**
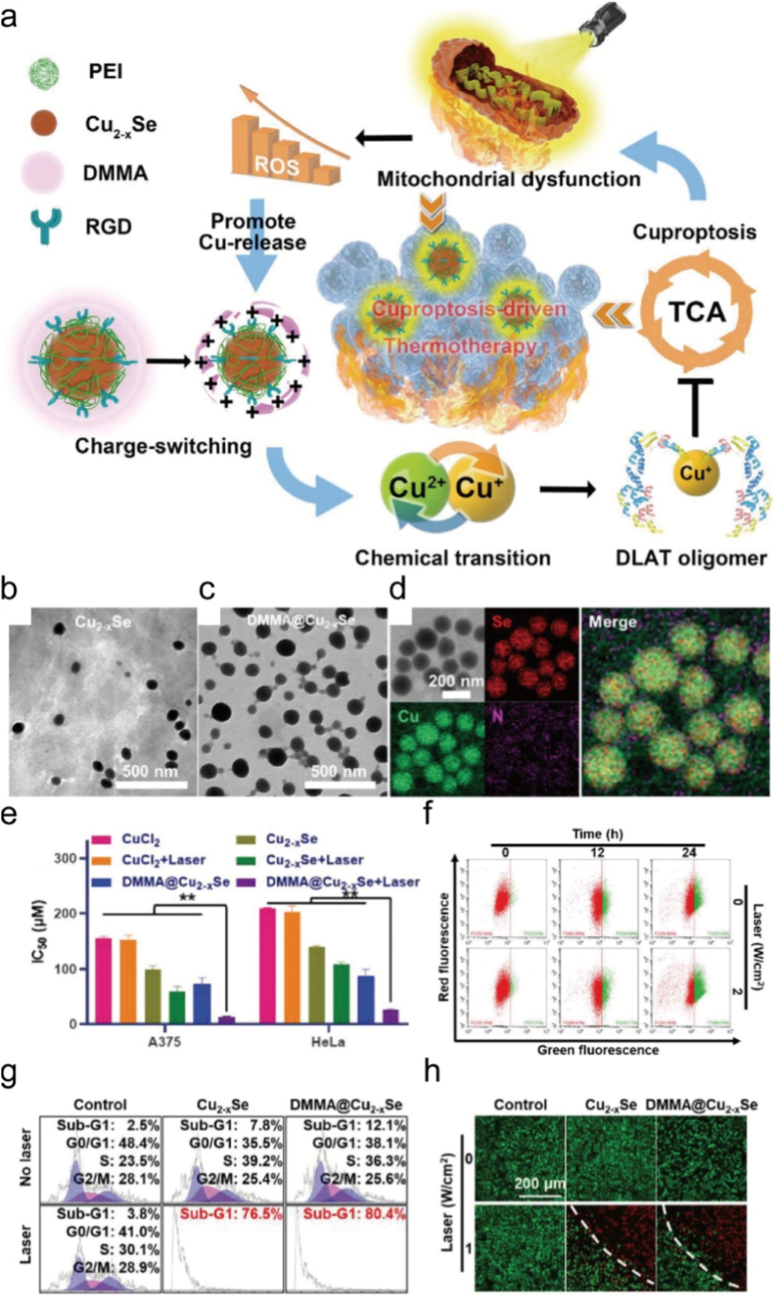
(a) Schematic depiction of DMMA@Cu_2_-xSe harnessing cuproptosis to amplify thermotherapeutic efficacy. (b) TEM image of Cu_2_-xSe. (c) TEM image of DMMA@Cu_2-x_Se. (d) HR-TEM and mapping image of DMMA@Cu_2-x_Se. (e) Cytotoxicity of CuCl_2_, Cu_2-x_Se and DMMA@Cu_2-x_Se either individually or combined with 808 nm laser against A375 and HeLa tumor cells. (f) Flow cytometry of mitochondrial membrane potential in A375 cells treated with 120 μM of DMMA@Cu_2-x_Se and 808 nm laser. (g) Cell cycle changes of A375 cells after treatment with 40 μM of Cu_2-x_Se and DMMA@Cu_2-x_Se and 808 nm laser. (h) Dying cell staining image of A375 cells incubated with 240 μM of Cu_2-x_Se and DMMA@Cu_2-x_Se either individually or combined with 808 nm laser. Reproduced with permission [279]. Copyright 2023, Wiley.

PTT uses NIR light to activate photosensitizers, generating local high temperatures that not only directly ablate tumor tissue but also trigger the controllable release of copper ions, creating a unique “thermal-ion therapy” synergistic effect [273]. NIR light has the ability to penetrate deep into tissues and can precisely activate nanomaterials loaded with copper precursors [274]. When photothermal conversion materials absorb light energy, the local temperature rapidly rises to 42-45 °C [275]. This mild thermal effect changes the permeability of tumor cell membranes and promotes the dissociation and release of Cu^2+^ from copper based nanoparticles [276]. This spatiotemporally controlled release avoids the systemic toxicity associated with conventional metal ion therapy [277]. Importantly, cuproptosis and the photothermal effect create a mutually reinforcing cycle. The heat produced by PTT speeds up the Fenton reaction of Cu^+^, and the Cu^2+^ formed in this process further blocks the production of iron-sulfur cluster proteins. This inactivates key enzymes in the mitochondrial TCA and causes the mitochondrial membrane potential to collapse [278]. At the same time, the photothermal effect causes damage that works together with copper-induced oxidative stress. This positive feedback loop of copper release, ROS increase, mitochondrial damage and more copper release, breaks the redox balance inside tumor cells. Chan et al. designed DMMA@Cu_2-x_Se, a programmed nanosystem for precise copper delivery and tumor targeting (Fig. 12a). In the acidic TME, DMMA detaches to expose PEI, flipping surface charge and enhancing cellular uptake (Fig. 12b–h) [279]. Intracellularly, Cu_2-x_Se releases Cu^2+^, inducing cuproptosis, inhibiting mitochondrial respiration, and sensitizing cells to thermotherapy. Laser irradiation further boosts ROS, promoting copper release and reinforcing cuproptosis, achieving potent synergistic antitumor effects.

### 5.2 Cu-based components of combination treatment

#### 5.2.1 CuO-based nanomaterials

**Fig. 13:**
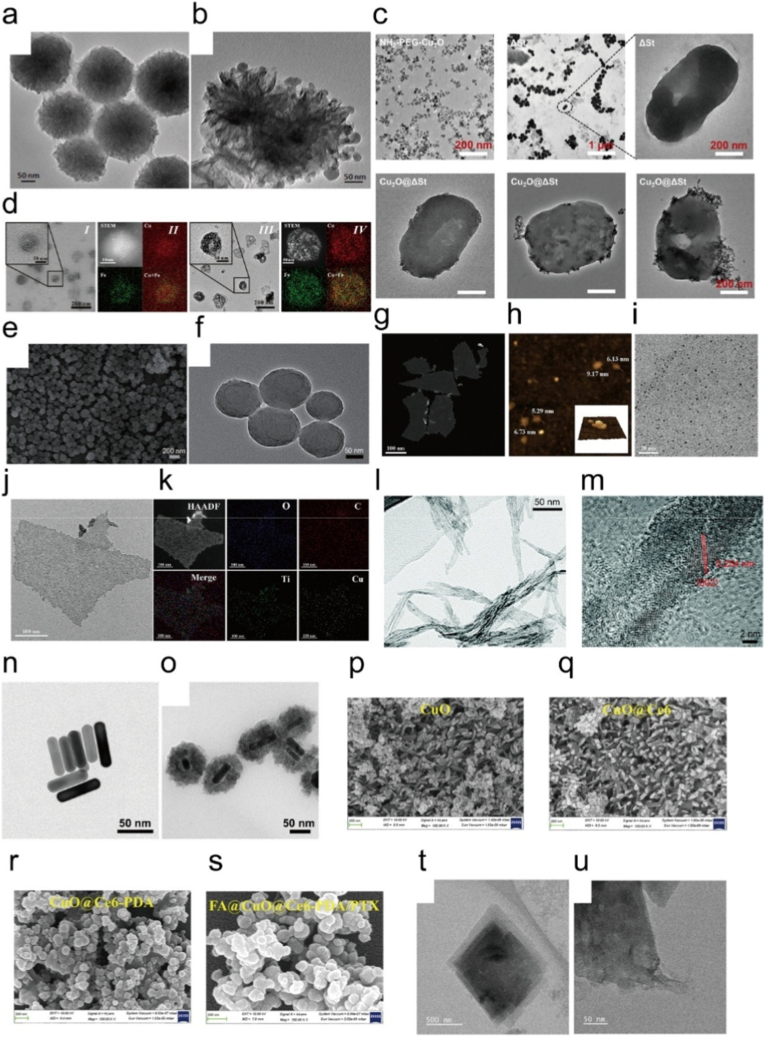
(a) TEM image of MoS_2_ nanoflowers. (b) TEM image of MoS_2_-CuO. Reproduced with permission [280]. Copyright 2021, ELSEVIER. (c) TEM images of Cu_2_O, ΔSt, and Cu_2_O@ΔSt. Reproduced with permission [281]. Copyright 2022, Springer Nature. (d) TEM and elemental mapping images of PDA:CuO_2_ (I and II) and ICPs@PDA:CuO_2_ NPs (III and IV). Reproduced with permission [282]. Copyright 2023, American Chemical Society. (e) SEM of CuO@CNSs. (f) TEM images of CuO@CNSs. Reproduced with permission [283]. Copyright 2020, Springer Nature. (g-h) TEM image and AFM image of Ti_3_C_2_ MXene nanosheets. i. TEM image of CuO_2_ nanodots. (j) TEM image of FMCG. (k) Mapping of FMCG. Reproduced with permission [284]. Copyright 2024, Wiley. (l) TEM image, and (m) HRTEM image of SiO_2_@CuO. Reproduced with permission [285]. Copyright 2020, Royal Society of Chemistry. (n) TEM images of Au NRs. o. TEM images of CuO_2_@Au NCs. Reproduced with permission [286]. Copyright 2024, Wiley. (p-s) FESEM analysis of synthesized different NPs with their corresponding EDX spectra. Reproduced with permission [287]. Copyright 2024, ELSEVIER. (t-u) TEM images of CZPCH. Reproduced with permission [288]. Copyright 2024, ELSEVIER.

In recent years, extensive efforts have been devoted to developing CuO-based nanoplatforms to potentiate the synergistic efficacy of PTT and PDT. Jiang et al. engineered MoS_2_-CuO heterostructures in which CuO catalyzed H_2_O_2_ into ·OH via Fenton like reactions while MoS_2_ mediated photothermal effects, achieving combined PTT/CDT and immune activation (Fig. 13a–b) [280]. Wu et al. reported Cu_2_O@ΔSt microbiotic nanomedicine, where bacterial metabolism converted Cu_2_O into CuS for tumor specific PTT and released Cu ^+^ for Fenton like CDT, thereby realizing PTT/CDT enhanced immunotherapy (Fig. 13c) [281]. Zhu et al. developed ICPs@PDA:CuO_2_ nanoparticles, in which CuO_2_ mediated ROS generation through Fenton like reactions and, together with PTT and chemotherapy, enabled programmed trimodal synergistic therapy (Fig. 13d) [282]. Similarly, Jiang et al. constructed CuO@CNSs-DOX nanoplatforms, where CuO improved photothermal conversion and released Cu^2+^ to promote ROS generation, while DOX provided chemotherapy, achieving integrated PTT/CDT/CT (Fig. 13e–f) [283]. Xiong et al. introduced FA@MXene/CuO_2_/GA nanocomposites, in which MXene mediated mild PTT, CuO_2_ released Cu^2+^ for Fenton like catalysis, and GA suppressed HSP90, collectively realizing synergistic mild PTT/CDT (Fig. 13g–k) [284]. Sun et al. designed SiO_2_@CuO nanotubes that functioned as both photosensitizers and photothermal agents, thereby enhancing PDT/PTT while amplifying ROS production via Fenton like processes (Fig. 13l–m) [285]. Hu et al. prepared an injectable CuO_2_@Au hydrogel capable of H_2_O_2_ self-supply and GSH depletion for CDT, with Au components enabling low temperature PTT, thus effectively preventing tumor recurrence and infection (Fig. 13n–o) [286]. Pal et al. developed FA@CuO@Ce6-PDA/PTX nanoparticles integrating CuO-mediated PTT, Ce6-driven PDT, CuO-induced ROS catalysis, and PTX chemotherapy, achieving trimodal combination therapy (Fig. 13p–s) [287]. Finally, Wang et al. constructed the Ce6@ZIF-8/PDA/CuO_2_/HA (CZPCH) nanoplatform, which self-supplied H_2_O_2_ and enhanced ROS generation through Fenton like reactions while combining PDT and PTT, ultimately realizing highly efficient CDT/PDT/PTT synergistic tumor therapy (Fig. 13t–u) [288].

#### 5.2.2 Cu_2-X_Se-based materials

**Fig. 14:**
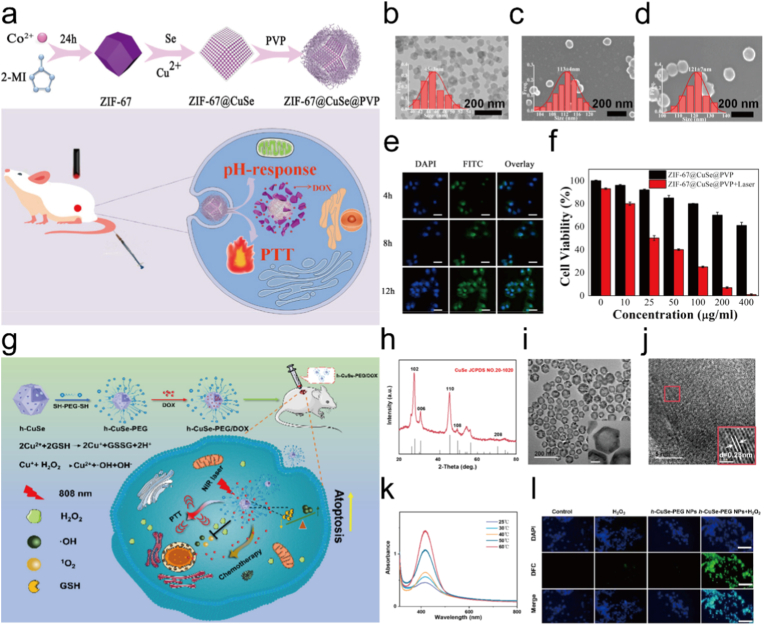
(a) Preparation of ZIF-67@CuSe@PVP nanoparticles. (b) Typical TEM image of ZIF-67. (c) Typical SEM images of ZIF-67@CuSe. (d) Typical SEM images of ZIF-67@CuSe@PVP. (e) Fluorescence images of 4T1 cells treated with DAPI and FITC-labeled ZIF-67@CuSe@PVP. (f) cell viability of 4T1 cells after incubation with or without 1064 nm irradiation of ZIF-67@CuSe@PVP. Reproduced with permission [289]. Copyright 2021, American Chemical Society. (g) Schematic Illustration of h-CuSe-PEG-Based theranostic platform for synergistic photo-enhanced CDT/PTT/Chemotherapy. (h) XRD pattern of h-CuSe NPs. (i) TEM image of h-CuSe NPs with an inset scale of 20 nm. (j) HRTEM image of h-CuSe NPs. (k) Effect of temperature on the Fenton-like activity of h-CuSe NPs. (l) CLSM images of 4T1 cells stained with ROS fluorescence probe DCFH-DA under different treatments. Reproduced with permission [290]. Copyright 2023, American Chemical Society.

Recent studies have highlighted the potential of Cu_2-X_Se-based nanoplatforms to enhance synergistic photothermal and photodynamic therapies. Wu et al. constructed ZIF-67@CuSe@PVP nanoparticles, which exhibited high photothermal conversion efficiency (36%) and pH-responsive doxorubicin release, thereby achieving potent chemo-PTT with significant antitumor efficacy (Fig. 14a–f) [289]. Although ZIF-67@CuSe@PVP contains a MOF-derived component, it is classified here as a Cu_2-X_Se-based system because Cu_2-X_Se serves as the primary photothermal and copper ion releasing component responsible for therapeutic synergy. Similarly, Li et al. developed hollow PEGylated CuSe nanoparticles (h-CuSe-PEG) with superior photothermal conversion efficiency (54.66%) for PTT, which not only depleted intracellular GSH but also catalyzed H_2_O_2_ to generate ·OH for CDT (Fig. 14g–l) [290]. Moreover, their hollow cavity enabled efficient doxorubicin loading, while PTT-induced hyperthermia further enhanced drug release, GSH depletion, and ROS generation, ultimately realizing combined CDT/PTT/chemotherapy with strong therapeutic outcomes. Together, these findings underscore the unique advantages of CuSe nanostructures in amplifying ROS production and photothermal effects, thereby significantly reinforcing the synergistic efficacy of PTT and PDT in tumor therapy.

#### 5.2.3 CuTe-based nanomaterials

**Fig. 15:**
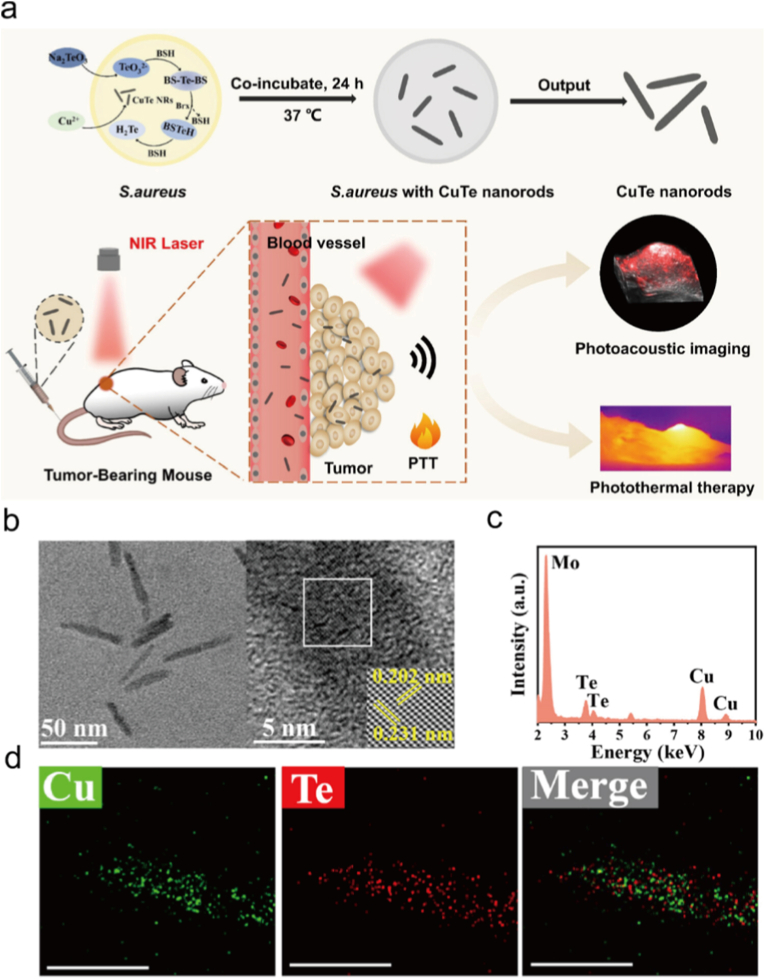
(a) Schematic diagram of cute nanorods synthesized by living *s. Aureus* cells for in vivo PAI and PTT. (b). TEM and HRTEM images of CuTe NRs. (c) EDS images of CuTe NRs. (d) HAADF-STEM images of CuTe NRs. Reproduced with permission [292]. Copyright 2024, American Chemical Society.

More advances have demonstrated that copper telluride (CuTe) nanostructures are promising agents for synergistic photothermal and photodynamic tumor therapy. Huang et al. emphasized in their review that CuTe, along with other copper chalcogenides, exhibits strong NIR absorption and high photothermal conversion efficiency, enabling effective PTT within safe laser power densities and offering enhanced therapeutic outcomes when integrated with photosensitizers or drugs for combined PTT/PDT [291]. Building on this, Zheng et al. reported the biosynthesis of CuTe nanorods using *Staphylococcus aureus*, which achieved remarkably high molar extinction coefficients and photothermal conversion efficiencie (Fig. 15a–d) [292]. These nanorods produced significantly stronger photoacoustic signals than indocyanine green and effectively suppressed tumor growth via PTT, highlighting their potential for NIR-II photoacoustic imaging–guided synergistic PTT/PDT. Together, these studies highlight the special advantages of CuTe nanomaterials in boosting photothermal effects and ROS based photodynamic responses, thus strengthening their use in multimodal therapy and diagnosis.

#### 5.2.4 CuS-based materials

Nowadays, CuS nanomaterials also have emerged as a powerful tool in phototherapy for tumor treatment, particularly excelling in enhancing both PTT and PDT (Table 3). Researchers have leveraged CuS's unique ability to convert NIR light into heat for PTT, while simultaneously generating ROS for PDT, creating a synergistic therapeutic effect [307]. For example, Yang et al. developed an intelligent nanoplatform where CuS not only provided photothermal effects but also triggered CDT through reactions with tumor produced H_2_S [308]. Similarly, Sheng et al. synthesized Ce6@CuS-Pt nanocomposites, utilizing CuS for PTT and photosensitizer Ce6 for PDT, further enhanced by Pt nanozymes to boost ROS generation [294]. Other studies have highlighted CuS's versatility, such as Qian et al.’s hydrogel system integrating CuS for PTT/PDT with pH-responsive drug release, or Wang et al.’s innovative CuS/Pt nanomotors that alleviated tumor hypoxia and improved PDT efficiency [293,295]. Additionally, Chen et al. demonstrated CuS's potential in NIR-II-guided PDT/CDT, where it enabled both photothermal effects and Fenton like reactions to generate hydroxyl radicals [22]. Collectively, these advances underscore CuS's dual role in overcoming tumor heterogeneity and resistance, offering a low toxicity, tunable platform for multimodal cancer treatment.

**Table 3:** Summary of the synergistic therapy combining CuS-based materials.

Core material	Tumor type	Outcomes	Ref
CuS + DOX@ZIF-8	4T1	self-healing/injectable and synergistic PTT/PDT/chemotherapy.	[293]
Ce6@CuS-Pt/PEG	CT26	superior to single mode phototherapy.	[294]
CuS/Pt (IR820)	4T1	chemical-NIR propulsion and augmented PTT/PDT.	[295]
AuNBP@CuS	4T1	triggers ICD.	[22]
HP-PCN@CuS	4T1	synergistic CDT/PDT/PTT.	[296]
CuCo_2_S_4_-Pt-PEG	4T1	T1-weighted MRI/PAI and suppresses metastasis via immune response.	[297]
Lipo@ICG@CuS	4T1	solves large NP penetration/small NP clearance dilemma.	[298]
CuS@COF	4T1	85.1% phototoxicity of PTT/PDT.	[299]
HMCuS/Pt/ICG@MnO_2_@9R-P201	H22	activates cGAS-STING immune pathway and suppresses recurrence.	[300]
_D_Cu_x_S	MDA-MB-231	tumor targeted via NC3S aptamer and enhances PTT/PDT by optimizing TME.	[301]
DMOF@MnCO@CuS@Hairpin	Huh-7 and HepG2	PTT/PDT/CDT/gas therapy synergy.	[302]
HA-CuS/MnO_2_	4T1	T1-weighted MRI guidance and synergistic CDT/PTT/PDT.	[303]
GOx@HCuS@HA	4T1	CD44-targeted (HA) and ST/PTT/PDT/CDT.	[304]
CuS/Ag/Pt/ICG/DOX	H22	Chemotherpay + PDT + PTT and TME remodeling via GSH depletion.	[305]
Gold star@PB@CuS	4T1	No gold passivation and broad NIR absorption (810 nm).	[306]

#### 5.2.5 Cu-MOF based materials

**Fig. 16:**
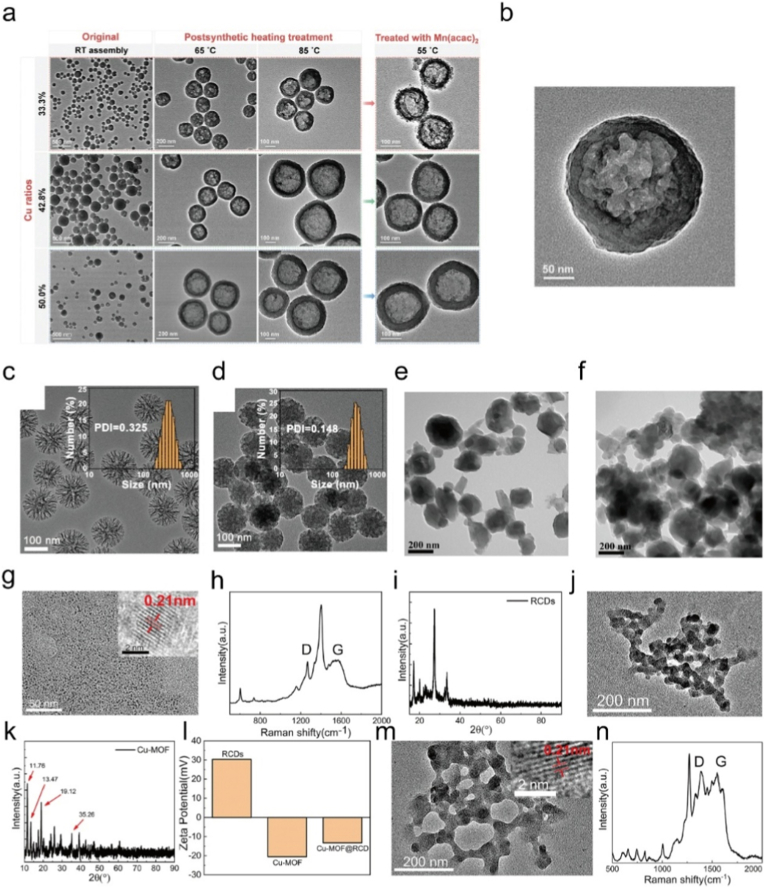
(a) TEM images of hollow Cu/Zn-MOFs formation with various Cu ratios at different treatment temperatures. (b) TEM image of hollow Cu/Zn-MOF after treated at 85 °C. Reproduced with permission [314]. Copyright 2021, Wiley. (c) TEM images of DMSNs. (d) TEM images of HDPI. Reproduced with permission [315]. Copyright 2022, American Chemical Society. (e) TEM image of PCN-224(Cu). (f) TEM image of PCN-224(Cu)@PDA. Reproduced with permission [311]. Copyright 2024, American Chemical Society. (g) The TEM images of RCDs. (h) Raman spectrum of RCDs. (i) XRD patterns of RCDs. (j) TEM images of Cu-MOF. (k) XRD patterns of Cu-MOF. (l) Zeta potential of RCDs, Cu-MOF, and Cu-MOF@RCD. (m) The TEM images of C-MOF@RCD. (n) Raman spectrum of Cu-MOF@RCD. Reproduced with permission [310]. Copyright 2023, Royal Society of Chemistry.

Many researches have highlighted the potential of Cu based MOFs as multifunctional nanoplatforms to synergistically enhance photothermal and photodynamic therapies (Table 4). Cheng et al. constructed a Cu/Zn MOF derived hollow porous nanocomposite capable of loading ICG, where NIR irradiation triggered both PTT and PDT, while Cu ions mediated Fenton like reactions to amplify ROS, thereby achieving a synergistic CDT/PDT/PTT effect (Fig. 16a–b) [314]. Similarly, Bian et al. reported a dendritic mesoporous silica system decorated with Cu-MOFs and ICG, in which Cu^2+^ released in the TME depleted GSH and catalyzed hydroxyl radical generation, while ICG mediated PTT/PDT further strengthened the oxidative stress for enhanced multimodal therapy (Fig. 16c–d) [315]. Zhang et al. designed a porphyrin based Cu-doped MOF coated with polydopamine, where the porphyrin core served as a photosensitizer for PDT, PDA provided PTT capability, and the Cu^2+^/Cu^+^ cycle initiated Fenton like reactions for CDT, enabling an efficient trimodal therapeutic strategy (Fig. 16e–f) [311]. In addition, Su et al. developed a carbon dot doped Cu-MOF that combined PTT and PDT with Cu mediated ROS amplification and GSH depletion, and when integrated with immune checkpoint blockade, this platform effectively eliminated both primary and metastatic tumors (Fig. 16g–n) [310]. Collectively, these studies demonstrate that Cu-MOF nanostructures not only provide intrinsic catalytic activity for ROS generation but also integrate photothermal and photodynamic effects, thus offering a powerful strategy for synergistic tumor ablation.

**Table 4:** Summary of the synergistic therapy combining Cu-MOF based materials.

Core material	Tumor type	Outcomes	Ref
HT@DMSNs-Pt(IV)@ICG	H22	PTT + PDT + CDT + chemotherapy	[309]
Cu-MOF@RCD	CT26	PDT + PTT + CDT + DG + ICB	[310]
PCN-224(Cu)@PDA	4T1	CDT + PDT + PTT	[311]
CuMoO_4_/g-C_3_N_4_	HepG2	CDT + PTT + PDT; CT + MRI	[312]
PCN-224@Au NPs@CP	CT26	PTT + PDT + CDT + cuproptosis	[313]
Ce6@ZIF-8/PDA/CuO_2_/HA	CHO + HepG2	CDT + PDT + PTT	[288]

### 5.3 Cuproptosis inducers

In the current field of tumor delivery research, to overcome the limitations of small molecule copper ionophores, including Elesclomol (ES), 8-Hydroxyquinoline (8HQ), Pyrithione, UM4118 and Disulfiram (DSF) with short circulation half-lives, poor tumor targeting, and systemic toxicity, researchers have developed a variety of intelligent nanoplatforms integrated with PTT or PDT effects [[316], [317], [318], [319]]. These platforms significantly amplify the therapeutic efficacy of cuproptosis through multimodal synergistic strategies. Specifically, the photothermal effect is often employed as a precise switch to trigger drug release. Nanoplatforms such as PEG@Cu_2_O-ES constructed by Wei Li et al. utilize localized hyperthermia generated by NIR to induce nanomaterial disintegration, thereby rapidly releasing copper ions and ES in the tumor [320]. By combining heat induced ROS to attack the ATP-Cu pump and inhibit copper efflux, these systems employ an increased influx, decreased efflux strategy to intensify intracellular copper overload. Meanwhile, the ROS generated by the photodynamic effect not only directly damage cells but also exhibit deep biochemical coupling with the cuproptosis pathway. Notably, the DSF/Ce6@ZIF-8@CuO_2_ cascade platform developed by Tong Li et al. alleviates the hypoxic microenvironment via self-supplied oxygen to enhance Ce6-mediated PDT [321]. The superoxide anions produced can directly reduce intracellular Cu^2+^ to the more pro-death active Cu^+^, thereby bypassing complex reduction processes to directly induce DLAT protein aggregation. Furthermore, this synergistic effect manifests in the complete disruption of tumor redox homeostasis. Cel-Cu NPs developed by Lu et al. and ZCA NSs investigated by Tang et al. drastically deplete GSH via copper ion mediation, significantly weakening the ability of cells to scavenge ROS generated by PDT/CDT [322,323]. This subjects cancer cells to a double attack of proteotoxic and oxidative stress. Such multidimensional cellular damage ultimately triggers robust ICD. As demonstrated by the AHPR developed by Jing et al., the release of DAMPs promotes dendritic cell maturation and increases CD8^+^ T cell infiltration, effectively remodeling cold tumors into hot tumors [324]. This opens up broad prospects for the combined application of immune checkpoint inhibitors.

## 6 Synergistic effects on calcium overload and phototherapy

The strategy of combining PTT or PDT with calcium overload significantly enhances tumor treatment efficacy through multimodal synergistic effects, with the core being the regulation of calcium ion homeostasis imbalance using light-controlled technology. PTT activates the transient receptor potential vanilloid 1 (TRPV1) to promote the influx of extracellular Ca^2+^ by heating generated from NIR excited nanomaterials, while the decrease in mitochondrial membrane potential impairs the buffering capacity of Ca^2+^, exacerbating intracellular calcium overload. PDT generates ROS to disrupt mitochondrial function and activates endoplasmic reticulum calcium channels to release endoplasmic reticulum Ca^2+^, further amplifying the calcium overload effect. Additionally, PTT or PDT can synergize with acid responsive materials to decompose and release exogenous Ca^2+^, and enhance the influx of extracellular Ca^2+^ through calcium ion carriers. This combined strategy forms a synergistic effect through multitarget attack and ICD-induced immune activation.

### 6.1 Mechanisms of calcium overload amplified phototherapy

#### 6.1.1 Calcium overload amplified PTT

Calcium overload, by disrupting intracellular Ca^2+^ homeostasis, leads to a decrease in mitochondrial membrane potential, impairment of ATP synthesis, and energy depletion, thereby inducing apoptosis or necrosis of tumor cells, which is an endogenous self-destruction amplification mechanism [325]. PTT can not only directly generate heat to kill tumors but also accelerate the decomposition of calcium based nanomaterials such as CaCO_3_ to release Ca^2+^, or activate ion channels such as TRPV1 to promote the influx of extracellular Ca^2+^, thereby exacerbating mitochondrial calcium overload [[326], [327], [328]]. Meanwhile, the unitive effect can also enhance the generation of ROS, forming a double blow with mitochondrial dysfunction caused by calcium overload, and further break through the self-protection mechanism of tumor cells in combination with autophagy inhibitors [329,330]. Therefore, the combination strategy of “PTT + calcium overload” not only overcomes the limitations of single PTT in efficacy and tolerance but also significantly improves the therapeutic effect on tumors, becoming a promising new multimodal treatment regimen. For instance, Liu et al. designed CaCO_3_@CQ@pDB nanoparticles, where NIR-II irradiation triggered strong photothermal heating by the conjugated polymer pDB, simultaneously inducing massive Ca^2+^ influx and mitochondrial damage. The released chloroquine further suppressed autophagy, abolishing tumor self-protection and thereby strengthening the combined PTT/Ca^2+^ overload effect [331]. Similarly, Wang et al. constructed the SA/Cur@CaCO_3_-ICG system, in which acidic TME accelerated Ca^2+^ release from CaCO_3_, disrupting mitochondrial membrane potential, while ICG mediated heating promoted additional Ca^2+^ release and ROS generation, leading to synergistic apoptosis [332]. In another study, Yu et al. employed CNQ nanoparticles incorporating CaCO_3_ and a photothermal agent to mildly activate the TRPV1 pathway, facilitating mitochondrial Ca^2+^ overload and dismantling tumor defenses under gentle hyperthermia [333]. Furthermore, Ca^2+^ nano modulators for breast cancer demonstrated that NIR irradiation not only enhanced photothermal ablation but also accelerated Ca^2+^-mediated energy depletion and apoptosis, yielding strong in vivo antitumor efficacy. Collectively, these studies confirm that calcium overload acts as a potent amplifier of PTT by disrupting intracellular Ca^2+^ homeostasis, impairing mitochondrial function, and blocking compensatory mechanisms, thus offering a promising dual hit strategy for improved tumor ablation.

#### 6.1.2 Calcium overload amplified PDT

Recent studies have demonstrated that calcium overload plays a pivotal role in enhancing the efficacy of PDT through multiple mechanisms. Upon light irradiation, photosensitizer mediated ROS generation disrupts intracellular calcium homeostasis, leading to excessive Ca^2+^ accumulation, and this calcium overload synergistically amplifies PDT induced cytotoxicity through multiple pathways [117,334,335]. First, Ca^2+^ influx into mitochondria collapses the mitochondrial membrane potential, triggers MPTP opening, and promotes cytochrome *c* release and caspase cascade activation, thereby enhancing apoptosis [18,[336], [337], [338], [339]]. Second, ROS mediated damage to endoplasmic reticulum Ca^2+^-ATPases such as SERCA2 causes massive ER Ca^2+^ efflux, initiating ER stress and caspase-12-dependent apoptotic signaling [[340], [341], [342]]. Third, ROS induced plasma membrane disruption or aberrant activation of ionotropic receptors facilitates extracellular Ca^2+^ entry, further exacerbating intracellular calcium overload [[343], [344], [345], [346]]. Under high PDT doses, sustained Ca^2+^ dysregulation also leads to osmotic imbalance, membrane rupture, and necrosis [[347], [348], [349]]. In addition, calcium-based nanomaterials that decompose under acidic TMEs can release Ca^2+^ and simultaneously cooperate with ROS to induce mitochondrial dysfunction and oxidative stress, thereby amplifying PDT efficacy and even suppressing metastasis [[350], [351], [352]]. For instance, Pang et al. designed calcium-enriched carbon nanoparticles loaded with indocyanine green (Ca-CNPs@ICG), which released Ca^2+^ in the TME while generating ^1^O_2_ under irradiation [337]. The synergistic effects of GSH depletion and Ca^2+^ overload induced mitochondrial membrane potential collapse, thereby amplifying PDT-induced apoptosis. Similarly, Hong et al. reported that Photofrin-mediated PDT in glioma cells elevated intracellular Ca^2+^ primarily from internal stores, which reduced cell adhesion and increased cytotoxicity, while calcium chelation attenuated these effects [353]. Ding et al. further demonstrated that HMME-PDT in HeLa cells induced rapid Ca^2+^ elevation and SERCA2 degradation, leading to ER Ca^2+^ release and caspase-12-dependent apoptosis, with calcium chelators partially rescuing cell survival [354]. More recently, Guo et al. developed a BPQD@CaO_2_-PEG-GPC3Ab nanoplatform in which CaO_2_ served as a Ca^2+^ reservoir and ROS source. Upon acidic activation and NIR irradiation, the platform simultaneously triggered Ca^2+^ overload, mitochondrial dysfunction, and enhanced ^1^O_2_ production, resulting in self-reinforcing PDT efficacy [341]. Collectively, these findings underscore that calcium overload, whether derived from mitochondrial uptake, ER release, membrane disruption, or exogenous calcium-based nanomaterials, acts as a powerful amplifier of ROS-mediated phototoxicity and provides a promising strategy to potentiate PDT against tumors.

### 6.2 Strategies of combination treatment

#### 6.2.1 CaCO_3_-based materials

**Fig. 17:**
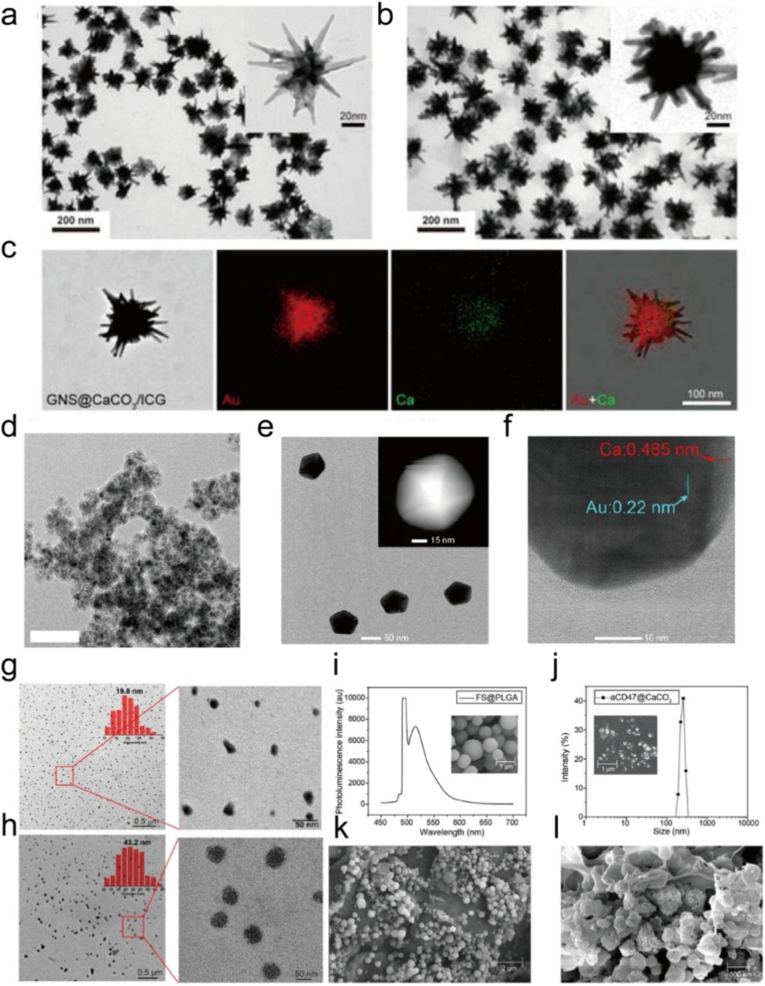
(a) TEM images of GNS. (b) TEM images of GNS@CaCO_3_/ICG. (c) STEM and EDS elemental mapping images of GNS@CaCO_3_/ICG. Reproduced with permission [355]. Copyright 2017, Lvyspring International Publisher. (d) TEM image of FPCI NPs. Reproduced with permission [356]. Copyright 2018, ELSEVIER. (e) TEM image, and (f) HRTEM image of PGP/CaCO_3_@IR820/DTX-HA. Reproduced with permission [357]. Copyright 2022, ELSEVIER. (g) HRTEM images of PDA-GSH-PEG5k, and (h) LST-IR820-CaNPs. Reproduced with permission [358]. Copyright 2023, American Chemical Society. (i) SEM images and fluorescence spectra of FS@PLGA. (j) SEM images and dynamic light scattering particle size distribution of aCD47@CaCO_3_. (k) SEM image of local magnification of APHP photothermal-photodynamic hydrogel and internal δ-ALA@PLGA microspheres. (l) Locally enlarged SEM image of CCCA immunomodulatory hydrogel and its internal aCD47@CaCO_3_. Reproduced with permission [359]. Copyright 2024, Wiley.

In recent years, numerous studies have confirmed that CaCO_3_, as an acid sensitive carrier in the TME, can stably load photosensitizers and drugs, and decompose to release Ca^2+^ under acidic conditions, inducing calcium overload, causing mitochondrial dysfunction and cell apoptosis, thereby forming synergy with photothermal and photodynamic effects. Liu et al. constructed a GNS@CaCO_3_/ICG nanoplatform in which the CaCO_3_ shell not only stabilized the photosensitizer ICG to prevent its deactivation during circulation but also decomposed in the acidic TME to release both ICG and Ca^2+^, thereby achieving tumor specific drug release and synergistically enhancing the antitumor efficacy of PTT and PDT through the photothermal properties of gold nanostars and the photodynamic effect of ICG (Fig. 17a–c) [355]. And Xue et al. developed Fe_3_O_4_@PDA@CaCO_3_/ICG nanocomposites that integrated the photothermal capability of polydopamine with the photodynamic activity of ICG. In this system, the CaCO_3_ coating not only improved the stability of ICG but also decomposed under acidic conditions to release Ca^2+^, inducing calcium overload and further promoting tumor cell apoptosis, which in turn reinforced the combined PTT/PDT effects (Fig. 17d) [356]. Tan et al. constructed a PGP/CaCO_3_@IR820/DTX-HA nanoplatform in which the CaCO_3_ shell decomposed in the TME to release the photosensitizer IR820, the chemotherapeutic agent docetaxel, and Ca^2+^, achieving synergistic inhibition of castration resistant prostate cancer through combined PTT/PDT/chemotherapy (Fig. 17e–f) [357]. Chen et al. designed LST-IR820-CaNPs, where the acid sensitive CaCO_3_ matrix released Ca^2+^ in the TME and simultaneously generated CO_2_ bubbles to provide ultrasound imaging signals. Together with the photothermal and photodynamic activities of IR820 and the tumor penetration enhancing effect of losartan, this nanoplatform achieved improved therapeutic depth and efficiency of PTT/PDT (Fig. 17g–h) [358]. Chen et al. further proposed a strategy of encapsulating CaCO_3_ nanoparticles, photosensitizers, and immune adjuvants into a postsurgical implantable hydrogel, which upon light irradiation enabled PDT/PTT and released Ca^2+^ to induce calcium interference therapy (CIT), thereby promoting ICD, enhancing immune activation, and effectively preventing recurrence and metastasis of oral cancer (Fig. 17i–l) [359]. In summary, calcium overload triggered by CaCO_3_ not only provides a unique endogenous synergistic mechanism for PTT/PDT but also paves a new direction for multimodal antitumor strategies.

#### 6.2.2 CaP-based materials

Calcium phosphate (CaP) has become a key material for enhancing the efficacy of PTT and PDT due to its good biocompatibility, pH-responsive degradation, and Ca^2+^ regulation capabilities. Significant progress has been made in related research, ranging from theoretical reviews to the design of specific nanoplatforms. Wang et al. first clarified through a review that CaP is an important category of exogenous Ca^2+^ overload nanomaterials, systematically summarizing the research progress on the combination of mitochondrial Ca^2+^ overload with PTT, PDT, and other therapies, laying a theoretical foundation for the application of CaP in phototherapy [117]. In terms of constructing specific nanoplatforms, Liu et al. designed an ICG/CaP@GOX-CAT@SA nanosystem, in which CaP not only serves as a carrier for the photosensitizer ICG, but also has glucose oxidase and catalase co-immobilized on its surface [360]. These enzymes can convert glucose in the TME into oxygen through a cascade reaction, effectively alleviating the hypoxia dilemma faced by PDT and ultimately achieving synergistic enhancement between starvation therapy and PDT. Zhu et al. developed a MICaP monolayer hydroxide nanosheet that utilizes the pH-responsive degradation characteristics of Ca_3_(PO_4_)_2_ to release excess Ca^2+^, inducing mitochondrial membrane potential damage in tumor cells and reducing oxygen consumption in the TME [361]. Meanwhile, ICG under NIR induces apoptosis through PDT by generating ^1^O_2_ and necrosis through PTT, further activating ICD, forming a multidimensional synergy between PDT/PTT/immunotherapy, with the release of Ca^2+^ from CaP being the core link of this synergistic effect. In addition to its carrier function, CaP can also enhance the efficacy of phototherapy by regulating TME metabolism or ion valence states. Zhang et al. constructed an MPCF nanoplatform with porous CaP as the carrier encapsulating iron dextran, which releases its payload in the acidic TME, combined with polydopamine (PDA)-mediated PTT to achieve enhanced combination of CDT and PTT [362]. In Xu et al.’s polydopamine-CaP composite nanomedicine, the calcium efflux inhibitor curcumin (Cur) released from the degraded CaP shell, together with Ca^2+^, disrupts mitochondrial metabolism and synergizes with GOx's glycolysis inhibition to reduce ATP levels and downregulate the expression of HSPs, significantly improving PTT sensitivity [363]. Yan et al. used CaP to seal DNAzyme (MNAzyme) and the PTT dye IR780 in RGD-modified DSPE-PEG micelles [328]. Under acidic conditions, CaP degrades to release MNAzyme and Ca^2+^, while Cur maintains a high intracellular Ca^2+^ level to disrupt mitochondrial Ca^2+^ homeostasis and further inhibit HSPs function, achieving triple sensitization of PDAC to PTT. Li et al. ’s Mn-doped CaP nanoclusters (MnCaP NCs) use CaP as a biocompatible matrix and take advantage of the valence conversion characteristics of Mn^2+^/Mn^4+^ [364]. After PTT, Mn^4+^ is reduced to Mn^2+^ by GSH, simultaneously enhancing MRI imaging and CDT effects, constructing a PTT-CDT sequential treatment system. Wang et al. designed TPP modified POM/CaP nanospheres (TPC/2DG NSs) also rely on CaP's controlled release capability to deliver the glycolysis inhibitor 2DG, which synergizes with mitochondrial oxidative damage to reduce ATP production and inhibit HSPs synthesis to enhance PTT efficacy [365]. It is worth noting that CaP also shows unique value in the precise monitoring of PDT. In summary, through multiple mechanisms such as carrier mediation, Ca^2+^ regulation, metabolic intervention, and monitoring assistance, CaP has significantly enhanced the therapeutic effects and synergistic potential of PTT and PDT, providing diversified strategies for the clinical translation of tumor phototherapy.

#### 6.2.3 CaO_2_-based materials

In the context of advancing CaO_2_ augmented PTT and PDT for cancer, a series of innovative nanoplatforms have been developed in recent years. As early as 2021, Yan et al. constructed the MCMnH + CaO_2_ nanosystem, where CaO_2_ collaborated with MnO_2_ to dual channel alleviate tumor hypoxia and amplify ROS generation, while melanin nanoparticles within the platform further promoted photosensitizer mediated ROS production under NIR irradiation, ultimately enabling combined antitumor effects of PDT, PTT, and CDT [366]. Subsequently, Pang et al. constructed the N-CNS-CaO_2_-HA/Ce6 NCs nanoplatform, where CaO_2_-HA NPs enable O_2_/H_2_O_2_ self-supply to optimize TME for tumor specific CDT and PDT, while N-CNSs act as PTT/CDT agents and Ce6 carriers to achieve PTT/CDT/PDT synergy [367]. Most recently, Liang et al. developed phototherapeutic liposomes preloaded with ICG, CaO_2_, and L-BSO. Under NIR laser irradiation, the liposomes released hyaluronidase to degrade the tumor extracellular matrix and improve drug penetration, while CaO_2_ alleviated TME hypoxia and depleted GSH to boost PDT/PTT effects, thereby achieving efficient synergistic suppression of breast cancer [368]. Collectively, these studies highlight CaO_2_'s versatile role in addressing TME limitations to reinforce PDT/PTT synergy, providing valuable insights for the design of next generation tumor phototherapeutic nanoplatforms.

#### 6.2.4 Calcium overload inducers

**Fig. 18:**
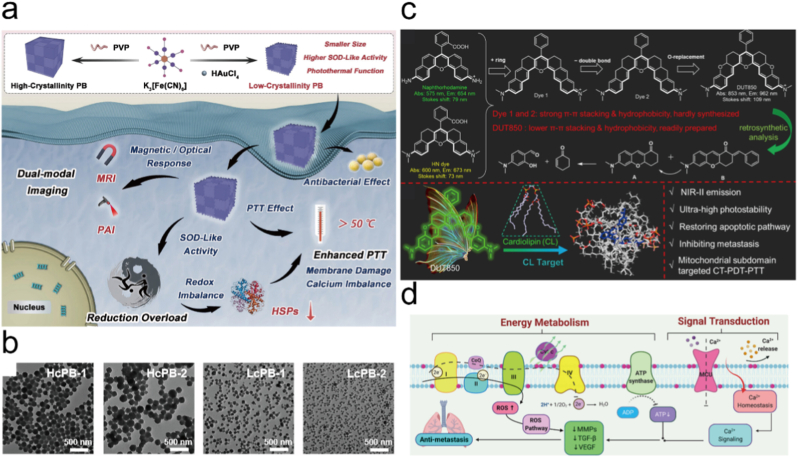
(a) The synthesis process of Prussian blue nanosystem, imaging properties, disruption of redox balance, antibacterial effect, and antitumor properties. (b) TEM images of all samples. Reproduced with permission [369]. Copyright 2025, Wiley. (c) Schematic illustration of molecular design and chemo-PTT-PDT of CL-Targeted DUT850. (d) Schematic diagram of DUT850-induced antitumor metastasis mechanisms. Reproduced with permission [370]. Copyright 2022, American Chemical Society. (For interpretation of the references to colour in this figure legend, the reader is referred to the Web version of this article.)

Significant progress has been made in the field of tumor phototherapy regarding calcium overload to enhance PTT and PDT. Non-exogenous calcium intake has also been extensively studied by many researchers. Yao et al. proposed a regulation scheme based on tetrachloroauric acid (HAuCl_4_) and prepared low crystallinity Prussian blue nanoparticles (LcPB NPs) of small size (Fig. 18a–b) [369]. These nanoparticles have high superoxide dismutase activity, which can induce tumor cell reduction overload. The redox imbalance mediated downregulation of HSPs not only effectively inhibits tumor growth through the inherentPTT mode of LcPB NPs but also disrupts cellular calcium homeostasis. Moreover, its inhibitory effect on symbiotic bacteria further enhances the overall anticancer effect. Under another strategy, Bian et al. designed the cardiolipin targeted NIR-II fluorophore DUT850 and its bovine serum albumin encapsulated form DUT850@BSA. DUT850, with a rigid V-shaped backbone, positive charge, and lipophilicity, can specifically recognize and efficiently bind to CL. This binding induces physiological disruptions including Ca^2+^ overload, along with excellent PDT and PTT performance. The Ca^2+^ overload enhances the phototoxicity of DUT850 toward cancer cells under safe 808 nm laser irradiation, and DUT850@BSA exerts a synergetic chemo-PDT-PTT effect on the 4T1 tumor mouse model, ultimately achieving solid tumor annihilation and metastasis inhibition, which can be real time monitored via the NIR-II fluorescence of DUT850 (Fig. 18c–d) [370]. The above studies all take calcium homeostasis regulation as the key entry point and achieve the synergy of calcium overload and PTT through the design of different nano drugs, providing a new paradigm for the efficient treatment of refractory tumors.

## 7 Other metal ions

### 7.1 Magnesium ions

Magnesium ions and their derivatives, such as magnesium oxide (MgO), magnesium peroxide (MgO_2_), and magnesium coordination compounds exhibit multidimensional roles in enhancing tumor PDT [149]. Their core mechanisms revolve around alleviating the tumor hypoxic microenvironment, optimizing the delivery and activity of photosensitizers, blocking the energy metabolism pathways of tumor cells, and synergistically amplifying the ROS effect [329]. Currently, no explicit reports on magnesium ions directly enhancing PTT have been found in existing literature, and their functions are mainly focused on improving PDT efficacy and optimizing treatment safety.

#### 7.1.1 MgO/MgO_2_-based materials

Numbers of advances have highlighted the potential of magnesium-based nanomaterials, particularly MgO and MgO_2_, in enhancing PTT and PDT. The unique properties of MgO enable it to function as a mitochondrial electron transport chain inhibitor, thereby impairing oxidative phosphorylation and sensitizing tumor cells to ROS mediated oxidative stress. For example, Zhang et al. developed an MgO-ICG suspension (MgO-ICG@S), in which MgO not only blocked ETC function but also acted as a carrier for ICG [371]. Upon NIR irradiation, ICG produced abundant ROS, while MgO induced ETC disruption further amplified oxidative damage, resulting in highly efficient PDT against triple negative breast cancer. In addition, MgO_2_ has been explored as a TME responsive H_2_O_2_ self-supplying agent. Xie et al. constructed hyaluronic acid modified MgO_2_/Pd nanocomposites (MgO_2_/Pd@HA), which decomposed under acidic and hyaluronidase rich conditions to release H_2_O_2_ [372]. The in situ H_2_O_2_ was catalyzed by Pd nanoparticles into ·OH while consuming intracellular GSH, thereby inducing ferroptosis. Meanwhile, Pd endowed the system with strong NIR-II photothermal conversion, achieving synergistic PTT and ferroptosis mediated tumor inhibition. Collectively, these studies demonstrate that MgO-based nanoplatforms can potentiate PTT and PDT through multiple mechanisms, including mitochondrial inhibition, ROS amplification, ferroptosis induction, and immune modulation, offering promising strategies for tumor therapy.

#### 7.1.2 Magnesium silicate-based materials

Magnesium silicate (MgSiO_3_) has recently attracted attention as a multifunctional nanoplatform for enhancing PTT and PDT. Owing to its lamellar silicon oxygen tetrahedral structure, MgSiO_3_ exhibits high capacity for loading photosensitizers and chemotherapeutic agents through electrostatic interactions, while its structural responsiveness to acidic TME and hyperthermia allows for controlled drug release. Mao et al. constructed an MgSiO_3_ fiber membrane scaffold (MSFM) coloaded with ICG and DOX [140]. Upon NIR irradiation, ICG endowed the scaffold with excellent photothermal conversion efficiency, and the combined thermal effect and acidic TME triggered DOX release. This synergistic PTT-chemotherapy approach significantly suppressed osteosarcoma growth in vitro and in vivo, while simultaneously promoting bone regeneration, and highlights that MgSiO_3_-based systems not only potentiate photothermal and photodynamic effects through efficient photosensitizer delivery and drug release, but also modulate the TME, offering promising strategies for multimodal cancer therapy.

#### 7.1.3 Mg-based complexes

Mg-based complexes, particularly those involving porphyrins, phthalocyanines, and porphyrazines, have demonstrated great potential in enhancing PTT and PDT. By coordinating with magnesium ions, these photosensitizers exhibit improved photophysical and photochemical properties, including enhanced solubility, increased photostability, and elevated singlet oxygen quantum yields, which collectively augment ROS production during PDT. For example, Guney et al. synthesized thiol functionalized Mg(II) phthalocyanine derivatives (THL-MgPc), which displayed strong light absorption, high photostability, and significant singlet oxygen quantum yields (ΦΔ = 0.41-0.46), underscoring their potential as efficient PDT photosensitizers [373]. Similarly, Pinheiro et al. reported that the coordination of Mg(II) with octakis(trifluoromethylphenyl)-porphyrazine significantly boosted singlet oxygen generation and selective phototoxicity, yielding nanomolar IC50 values against A431 epidermal carcinoma cells upon red light irradiation, thus enhancing PDT efficacy and tumor selectivity [374]. In another study, Tarakanov et al. developed low symmetry A_3_B-type diazepinoporphyrazine Mg(II) complexes, which exhibited an anti-Kasha effect that facilitated additional triplet state pathways, thereby increasing singlet oxygen generation [375]. These Mg complexes formed stable polymeric nanoparticles with polyvinylpyrrolidone and showed pronounced phototoxicity toward MCF-7 breast cancer cells under red light. Collectively, these findings demonstrate that magnesium coordination complexes can effectively enhance PDT through improved photosensitizer stability, ROS generation, and tumor selectivity, offering promising strategies for clinical translation in light-based cancer therapy.

#### 7.1.4 Other Mg-based materials

Other Mg-based materials, including magnesium ion layered double hydroxides (Mg-Fe LDH), magnesium ferrites (MgFe_2_O_4_), magnesium alloys, and magnesium fluoride (MgF_2_), have recently been explored as versatile platforms for enhancing PTT and PDT. Owing to their ion exchange capacity and structural responsiveness, Mg-Fe LDHs can efficiently load chemotherapeutic drugs and photosensitizers, while releasing Mg^2+^ and Fe^2+^ under acidic or hyperthermic conditions to trigger Fenton like reactions, thereby amplifying ROS generation and reinforcing PDT. For instance, Li et al. constructed a thermo-sensitive hydrogel incorporating doxorubicin loaded Mg-Fe LDH, where mild hyperthermia promoted drug release and achieved synergistic PTT-chemotherapy against osteosarcoma, while simultaneously facilitating bone regeneration [141]. In another approach, Pacheco et al. developed elastic liposomes containing Mg_0.75_Ca_0.25_Fe_2_O_4_ ferrite nanoparticles coupled with gold nanorods, in which the magneto-plasmonic hybrid system exhibited strong NIR absorption and efficient photothermal conversion, enabling effective tumor ablation by PTT [148]. Magnesium alloys have also been engineered as photothermal platforms. Zhu et al. designed a montmorillonite/emodin/polydopamine modified magnesium alloy (MMT/Em/PDA), which showed photothermal conversion and NIR/pH-responsive drug release, thereby integrating PTT and chemotherapy [376]. This strategy not only disrupted mitochondrial function and downregulated PI3K-AKT and EMT signaling, but also promoted M_1_ macrophage polarization, enhancing antitumor immunity. Moreover, MgF_2_ has been employed as a photonic coating to improve light trapping and broadband absorption. Zhang et al. reported an MgF_2_/ZnS coated Ge/Cr multilayer absorber that achieved ultra broadband infrared absorption and efficient thermal conversion, demonstrating potential applications in PTT [377]. In short, these studies indicate that Mg-Fe LDH, MgFe_2_O_4_, Mg alloys, and MgF_2_ contribute to the enhancement of PTT/PDT through diverse mechanisms, including drug/PS delivery, ROS amplification, immunomodulation, and photonic optimization.

### 7.2 Manganese ions

#### 7.2.1 MnO_x_-based materials

MnO_x_-based compounds have emerged as multifunctional agents to amplify the efficacy of PTT and PDT through multiple mechanisms, including ROS generation, GSH depletion, hypoxia alleviation, and immune activation. Wang et al. designed a MnO_2_-shelled CaO_2_ nanoreactor (CaO_2_/MnO_2_-Ce6-PEG), where the MnO_2_ layer depleted GSH and generated O_2_ to relieve hypoxia, while the CaO_2_ core released H_2_O_2_, collectively inducing a ROS storm that boosted both PDT and CDT [378]. Ma et al. reported ICG/MnO_2_-HFn-mPEG-DSPE liposomes, in which MnO_2_ improved the oxygen supply under hypoxic conditions, thereby increasing the singlet oxygen quantum yield and enhancing ICG-mediated PDT efficacy [379]. Lu et al. constructed SQ-580@MnO_2_ nanoparticles, where MnO_2_ consumed intracellular GSH and released Mn^2+^ to catalyze hydroxyl radical production, while SQ-580 acted as a type I photosensitizer, thereby combining PDT, CDT, and ferroptosis to amplify ICD [380]. Moreover, Mu et al. reported FA-IR780/EGCG@MnO_2_ nanoagonists, which integrated mitochondria targeted PTT with Mn^2+^-mediated STING pathway activation, facilitating dendritic cell maturation and T cell infiltration for durable antitumor immunity [381]. Collectively, these studies highlight that manganese oxide based nanoplatforms enhance light triggered therapies through synergistic mechanisms of ROS amplification, hypoxia modulation, redox balance disruption, and immune stimulation, thereby overcoming the limitations of single modal PDT or PTT and offering great promise for clinical translation.

#### 7.2.2 Manganese salt-based materials

Manganese salts and manganese coordination compounds have been widely explored as multifunctional agents to enhance PTT and PDT owing to their redox activity, oxygen modulation, and catalytic properties. Wu et al. constructed MnCO_3_-based nanocubes (Mn-ER-Cy), which degraded in the acidic TME to release Mn^2+^, thereby catalyzing Fenton like reactions for hydroxyl radical generation while simultaneously mediating PDT and PTT [382]. This triple modal strategy induced excessive endoplasmic reticulum stress and pyroptotic cell death, amplifying antitumor immunity. Beyond salts, coordination complexes of manganese also demonstrate potent PDT effects. For example, Bora et al. reported Mn(III) porphyrins (Mn1-Mn5) as effective photosensitizers that generated singlet oxygen under visible light, with Mn(III)/Mn(II) redox cycling amplifying ROS production [383]. Among them, Mn(III)-ClTPP exhibited superior photocytotoxicity with negligible dark toxicity. Mn(III)-ClTPP is discussed here as a manganese coordination complex rather than a framework material, as it does not form an extended MOF architecture and primarily functions through molecular-level Mn-mediated redox modulation. Similarly, Yang et al. developed a Mn-based phycocyanin nanocomplex (PC@Mn), where Mn coordination improved tumor accumulation and retention, while phycocyanin served as a natural photosensitizer [384]. Under laser irradiation, the system significantly amplified PDT efficacy and enabled MRI guided therapy. Collectively, these studies highlight that manganese salts can release Mn ions to catalyze Fenton like reactions and relieve hypoxia, while manganese coordination compounds function as efficient photosensitizers or catalytic enhancers, thereby synergistically boosting PTT and PDT efficacy.

#### 7.2.3 Mn-MOFs

Mn-MOFs have attracted increasing attention in PTT and PDT owing to their unique redox activity, oxygen modulation capacity, and structural versatility for drug and photosensitizer delivery. By exploiting the Mn^2+^/Mn^4+^ redox cycle, Mn-MOFs can catalyze Fenton like reactions in the TME, thereby generating hydroxyl radicals to amplify ROS-mediated PDT. Additionally, the decomposition of MnO_2_ within Mn-MOFs alleviates tumor hypoxia and depletes GSH, reducing ROS scavenging and further enhancing PDT efficacy. For instance, Cao et al. engineered a MnO_2_-containing MOF system, in which glucose oxidase catalyzed glucose to produce H_2_O_2_ that subsequently reacted with MnO_2_ to generate O_2_, thus overcoming hypoxia and activating porphyrin photosensitizers through chemiluminescence to achieve light free PDT [385]. Yang et al. constructed a hybrid Mn-based MOF (Fe-TCPP@MnO_2_@JUG@HA), where the MnO_2_ shell decomposed H_2_O_2_ into O_2_ while consuming intracellular GSH, thereby boosting PDT and CDT synergistically [386]. The release of juglone further introduced a chemotherapeutic effect, realizing a triple modality therapy. In short, these studies highlight that Mn-MOFs enhance light triggered therapies by catalyzing ROS production, modulating the TME, synergizing with PTT, and serving as versatile carriers for photosensitizers and drugs, thereby overcoming the limitations of single modal therapy and demonstrating great promise for multimodal cancer treatment.

### 7.3 Zinc ions

#### 7.3.1 Zinc oxide-based materials

Zinc oxide (ZnO)-based nanomaterials have emerged as promising multifunctional platforms for enhancing PTT and PDT owing to their unique optical properties, efficient ROS generation, pH-responsive Zn^2+^ release, and biocompatibility (Table 5). For instance, Joe et al. constructed triphenylphosphonium functionalized gold nanorod/ZnO core–shell nanocomposites (CTPP-GNR@ZnO), which simultaneously achieved mitochondrial targeted PTT and PDT through efficient photothermal conversion and ROS generation under 780 nm irradiation [387]. Cai et al. developed UCNPs@mSiO_2_@ZnO@PPy nanocomposites, in which upconversion luminescence activated ZnO nanodots to produce ROS for PDT, while the PPy shell induced photothermal effects, leading to potent synergistic tumor eradication in vitro and in vivo [388]. Li et al. reported ZnO@DOX/ICG-LMHP nanoparticles, in which ZnO decomposed into Zn^2+^ in acidic TMEs to trigger the release of ICG and DOX, while also generating O_2_ and ROS for PDT and producing heat for PTT, thereby integrating chemotherapy, phototherapy, and immunotherapy into one nanoplatform [389]. To further improve light penetration and ROS yield, Dong et al. designed Au@ZnO heterostructures doped with graphene quantum dots (AZGH), which facilitated efficient electron hole separation and hot electron injection, enabling NIR-driven PDT and PTT for effective triple negative breast cancer treatment [395]. In addition, ZnO has also been integrated with scintillators and upconversion nanoparticles to overcome the limitations of shallow light penetration. Zhang et al. combined Ce-doped LiYF_4_@SiO_2_@ZnO, where radiation induced UV emission activated ZnO to generate hydroxyl radicals for oxygen independent PDT synergized with radiotherapy [391]. Li et al. further designed NaErF_4_@ZnO upconversion nanoparticles, where NIR-excited UCNPs sensitized ZnO to produce ROS, effectively killing thyroid carcinoma cells under 980 nm light [393]. Moreover, Zhang et al. proposed an intelligent H_2_O_2_-responsive LCL/ZnO nanodelivery system, which normalized tumor vasculature through ROS mediated TRPV4-eNOS signaling, thereby improving PDT efficacy and ameliorating the TME [394]. Collectively, these studies highlight that ZnO enhances PDT and PTT through multiple mechanisms including efficient ROS generation, photothermal synergy, Zn^2+^ mediated cytotoxicity, and heterostructure induced NIR responsiveness, offering a versatile and powerful strategy for multimodal cancer therapy.

**Table 5:** Summary of the synergistic therapy combining ZnO-based materials.

Core material	Tumor	Outcomes	Ref
CTPP-GNR@ZnO	CT-26	PTT + PDT	[387]
UCNPs@mSiO_2_@ZnO	U14	PTT + PDT + UCL + CT	[388]
ZnO@DOX/ICG-LMHP	4T1	PTT + PDT + ICD	[389]
Au@ZnO	HeLa + C2C12	PTT + PDT	[390]
SCNP@SiO_2_@ZnO-PEG	HeLa	PDT	[391]
NPs-ZnO	-	PDT	[392]
NaErF4@ZnO UCNPs	BHP 5-16	PDT	[393]
LCL/ZnO	MDA-MB-231, 4T1, NIH-3T3	PDT	[394]
ZnO-Ce6	4T1	PDT + Immunotherapy	[25]
ZnO@DOX/ICG-LMHP	4T1	PTT + PDT + Chemotherapy + Immunotherapy	[389]
CTPP-GNR@ZnO	CT-26	PTT + PDT	[387]
Au@ZnO@GQDs	4T1	PTT + PDT	[395]
Au@PEG-ZnO	LLC	SDT + PDT	[396]

#### 7.3.2 Zinc peroxide-based materials

Recently, ZnO_2_ has emerged as a pivotal building block for constructing “self-O_2_/self-ROS” nano sensitizers owing to its acid triggered decomposition into H_2_O_2_ and O_2_ together with synchronous Zn^2+^ release, endowing PTT and PDT with unprecedented amplification effects. Within the weakly acidic and H_2_O_2_-rich TME, the ZnO_2_ core undergoes an explosive breakdown that instantaneously elevates local O_2_ availability, directly relieving solid tumor hypoxia and multiplying ^1^O_2_ generation for PDT. The concurrently produced exogenous H_2_O_2_ subsequently feeds Fenton like or catalase mimicking reactions catalyzed by embedded metal centers, triggering a cascade conversion into ·OH or O_2_ and culminating in a ROS storm that overwhelms cellular antioxidant defenses. Meanwhile, the released Zn^2+^ penetrate mitochondria, suppress the activity of electron-transport-chain complexes I/III, and block both oxidative phosphorylation and glycolytic ATP synthesis, thereby undermining the tumor's ability to upregulate HSPs and GSH and softening its resistance to PTT/PDT. The synergistic Zn^2+^ overload and ROS burst further open the MPTP in a sustained manner, promoting cytochrome *c* efflux and amplifying apoptotic signaling that integrates photothermal and chemodynamic killing. Qiao et al. developed polydopamine encapsulated ZnO_2_ nanoparticles (ZnO_2_@PDA) that establish an intracellular Zn^2+^-H_2_O_2_ self-amplifying loop, disrupt the metabolism redox circuit, deplete ATP and GSH, and for the first time realize a trinity of dual starvation-oxidative stress mild PTT synergistic ablation [397]. Ren et al. fabricated DOX/ZnO_2_@Zr-Ce6/Pt/PEG nanocomposites that self-supply H_2_O_2_, catalytically generate O_2_ via Pt nanozymes, enhance ^1^O_2_ production by 2.94-fold, downregulate HIF-1α, and markedly sensitize combined chemo-PDT [398]. And Jin et al. constructed a BSA-ZnO_2_@CeO_2_-ICG cascade nanoreactor in which ZnO_2_ provides H_2_O_2_ substrates, CeO_2_ cycles between ·OH and O_2_ generation through dual enzyme mimetic activity, and ICG-mediated PDT under 808 nm irradiation produces a ROS storm, while Zn^2+^ overload induces mitochondrial dysfunction, achieving robust CDT/PDT synergy [399]. Collectively, ZnO_2_ furnishes a versatile “self-O_2_/self-ROS/metabolism blockade” triple mechanism that breaks through hypoxia-induced resistance and oxidative defenses, positioning itself as a central functional unit in next generation photo-chemo combinatory nano theragnostic.

#### 7.3.3 Zn-MOF based material

In the field of tumor phototherapy, zinc-based metal organic frameworks (Zn-MOFs) have emerged as core carriers for enhancing the efficacy of PDT and PTT, owing to their structural tunability, environmental responsiveness, and multifunctional integration capabilities. Relevant studies have developed a variety of high efficiency therapeutic systems and verified their excellent performance. For PDT enhancement, the two dimensional Zn-TCPP MOF constructed by Hang et al. enables performance regulation using the acidic TME [400]. It exhibits weak PDT efficacy under neutral conditions due to the degeneracy of the Q bands of TCPP. However, in an acidic environment, the MOF structure dissociates to release TCPP, and the PDT effect is significantly enhanced upon irradiation with a 660 nm laser, providing a novel strategy for controllable PDT. To address the limitation of tissue penetration depth in traditional PDT, Kan et al. developed the PME@Zn/Fe-ZIF-90-Lum composite system [401]. This system utilizes Fe^2+^ to catalyze the oxidation of endogenous H_2_O_2_ in tumors for luminol chemiluminescence, and activates the photosensitizer PME loaded in MOFs to generate ROS through chemiluminescence resonance energy transfer, enabling efficient antitumor PDT without the need for external light sources. In addition, Zn-MOFs also show significant synergistic effects in PTT enhancement. For example, the magnetic Nb_2_C/aptamer-PDA/ICG@Zn/CoMOF@1-MT/C (NTC) nanoreactor constructed by Bai et al. achieves dual targeting of tumors through AS1411 aptamers and magnetic effects [402]. Under laser irradiation, Nb_2_C and ICG synergistically generate a strong photothermal effect, causing a sharp temperature rise at the tumor site and inducing cell apoptosis. Simultaneously, it synergizes with the immunomodulators 1-methyltryptophan and metformin to achieve the combination of PTT, PDT, and immunotherapy. These studies fully confirm that Zn-MOFs can significantly enhance the therapeutic effects of PDT and PTT through mechanisms such as environment responsive release of photosensitizers/photothermal agents, targeted delivery, microenvironment regulation, and multitherapeutic mode synergy, providing diversified and efficient solutions for the phototherapy of tumors and infectious diseases.

#### 7.3.4 Zn^2+^-based complexes

Zn^2+^ complexes have emerged as highly versatile and effective agents for enhancing PDT, with growing potential in photothermal applications, through rational molecular design and innovative nano formulations. A primary strategy involves optimizing their photophysical properties to maximize singlet oxygen generation. For instance, Unlu et al. developed a phenanthroline substituted Zn phthalocyanine that achieves a remarkable singlet oxygen quantum yield (ΦΔ) of 0.98 under sono-photochemical activation, significantly surpassing its baseline performance [403]. To address the critical challenge of tumor hypoxia, Bonelli et al. engineered an Ir(III)-phthalocyanine conjugate encapsulated in redox responsive nano capsules, which enables efficient Type I/II ROS generation even in hypoxic environments, thereby expanding the therapeutic window [404]. Enhanced tumor targeting and cellular uptake are achieved through strategic functionalization, as demonstrated by Tarhouni et al. designed a triphenylphosphonium-substituted Zn phthalocyanine (TPP-ZnPc) that exhibits negligible dark toxicity and potent photoactivity at low concentrations [405]. Furthermore, Zn complexes serve as cores for advanced, multifunctional nanoplatforms that enable combination therapies. Zhang et al. constructed a chiral Zn-based nano assembly (Zn-UCMB) that uniquely depletes lactate in a nonoxygen dependent manner to reverse immunosuppression while simultaneously catalyzing H_2_O_2_ decomposition to self-supply oxygen, dramatically enhancing ICD [406]. For controlled delivery and stability, Yang et al. formulated a thermosensitive nanocomposite gel loaded with Zn phthalocyanine, which stabilizes the photosensitizer within a three dimensional network, reducing photobleaching to 16.9% and boosting singlet oxygen production by 50% [407]. Collectively, these advancements underscore the role of Zn complexes not only as superior photosensitizers but also as integral components of smart, mult mechanistic systems that overcome the inherent limitations of conventional phototherapies.

## 8 Conclusion and prospects

Despite the promising therapeutic outcomes demonstrated by these metal ion amplified phototherapeutic systems, several challenges remain to be addressed before clinical translation. The final section therefore discusses current limitations, safety considerations, and future perspectives.

### 8.1 Conclusion

Combining metal ions with PTT and PDT brings a major change to cancer treatment. This strategy goes beyond single mode therapies and leads to more flexible and efficient combination treatments. Its main advantage is that it can overcome the natural weaknesses of photothermal and photodynamic therapies used alone. Metal ions serve as strong auxiliary agents that can greatly enhance the therapeutic outcome of light-based therapy. The creation of intelligent nanoplatforms that respond to the TME enables precise control of ion release in space and time, maximizing the killing effect within tumors while limiting unwanted toxicity elsewhere. In essence, metal ion mediated therapy does not merely enhance phototherapy. It introduces a new concept that disturbs ionic balance and triggers multiple tumors suppressing mechanisms, including oxidative stress, metabolic disruption, and immune stimulation. And the reported metal ions amplified phototherapeutic systems share several overarching design principles that collectively govern their therapeutic performance. First, spatiotemporally controllable metal ion release represents a foundational requirement, typically achieved through TME responsive triggers or light activated mechanisms, ensuring localized ion accumulation while minimizing systemic toxicity. Additionally, effective coupling between phototherapy induced stress and MIDCD is essential, whereby photothermal heating or photodynamic ROS generation accelerates metal catalyzed redox reactions, mitochondrial dysfunction, or ion-overload, forming self-amplifying cytotoxic cascades. And rational nanomaterial engineering enables multifunctional integration, allowing simultaneous phototherapy, catalytic activity, imaging guidance, and immune modulation within a single platform, thereby enhancing therapeutic precision and robustness. However, biocompatibility, degradability, and clearance profiles emerge as critical considerations for translational relevance, emphasizing that therapeutic efficacy must be balanced with biosafety and controllability. Additionally, it should be noted that the limitations of phototherapy are not uniformly or completely resolved by MIDCD based strategies. Instead, MIDCD provides mechanistically complementary pathways that can partially compensate for these constraints under specific conditions. For instance, ferroptosis relies less on molecular oxygen than classical photodynamic processes, thereby maintaining cytotoxic activity in hypoxic tumor regions. Moreover, by lowering the cellular tolerance threshold to oxidative or thermal stress, MIDCD associated pathways enable effective tumor cell killing under milder irradiation conditions, potentially reducing off-target damage to surrounding normal tissues. Therefore, MIDCD should be viewed not as a universal solution to phototherapy limitations, but as a mechanistic amplifier that broadens the therapeutic window and adaptability of phototherapy in complex TMEs.

### 8.2 Discussion

Despite the encouraging therapeutic outcomes achieved with metal ion amplified phototherapeutic nanosystems, their further development and clinical translation are inevitably accompanied by a series of interconnected challenges that must be addressed in an integrated manner. A central concern lies in the difficulty of precisely regulating metal ion dosage and release kinetics, since uncontrolled or prolonged ion accumulation may lead to off-target toxicity and perturb systemic metal homeostasis, thereby narrowing the therapeutic window. At the same time, the efficacy of phototherapy itself is intrinsically limited by light penetration depth and tissue heterogeneity, which can compromise treatment performance in deep-seated or anatomically inaccessible tumors and thus place additional demands on material design. These issues are further compounded by the incomplete understanding of long-term biosafety, biodegradability, and clearance pathways of complex nanomaterials, particularly under conditions of repeated or high dose administration, raising legitimate concerns regarding chronic toxicity. From a translational standpoint, practical obstacles such as scalable synthesis, batch-to-batch reproducibility, and stringent regulatory evaluation also impose non-negligible barriers to clinical implementation. Within this context, the rational selection and engineering of metal ions become pivotal for achieving robust synergistic effects while mitigating risks, as their redox properties must be carefully matched to specific phototherapeutic modalities, favoring redox active ions for ROS dependent processes such as PDT or ferroptosis, while prioritizing signaling regulatory ions for stress sensitization and mitochondrial perturbation under photothermal conditions. Moreover, consideration of metal ion homeostasis and tumor specific dysregulation offers an opportunity to exploit intrinsic vulnerabilities of the TME, thereby enhancing selectivity and reducing systemic toxicity. This necessitates precise control over coordination chemistry and release behavior to ensure spatiotemporally confined ion liberation in response to light irradiation. Ultimately, the biosafety profile and metabolic fate of both the metal ions and their carrier systems remain decisive determinants of translational feasibility, underscoring the importance of dosage controllable, biodegradable nanosystems validated through rigorous safety assessments and clinically relevant models to bridge the gap between experimental promise and practical application.

### 8.3 Prospects

Although encouraging results have been achieved in preclinical studies, translating metal ion assisted phototherapy into clinical application still faces several important obstacles. Long term biosafety and the metabolic fate of metal containing nanomaterials remain major concerns, emphasizing the need for detailed toxicological evaluation and the design of biodegradable or excretable systems. Improving nanoparticle targeting efficiency to achieve higher accumulation in tumors is another key task, which may be addressed through optimized ligand modification and biomimetic coating strategies. From a technical viewpoint, the shallow tissue penetration of light, even in the NIR-II window, limits its use for deep or metastatic tumors. Therefore, combining ion delivery with other activation methods could provide a practical way to extend treatment depth and efficacy. Looking ahead, this research area holds substantial promise and will likely evolve through several important directions. A deeper understanding of how different metal ions influence distinct cell death pathways will guide more rational design of combination systems. The development of advanced “all-in-one” theragnostic platforms that couple real time imaging with feedback-controlled ion delivery will enable precise and patient tailored therapy. Furthermore, regulating ion channels and metabolic routes could offer strategies to eliminate cancer stem cells and counteract drug resistance, reducing the risk of relapse. The integration of metal ion mediated phototherapy with immunotherapy represents another exciting opportunity. Designing nanoplatforms that trigger ICD and simultaneously remodel the immunosuppressive TME could generate in situ vaccine like effects, leading to strong systemic anti-tumor immunity and long-lasting immune memory. Through close interdisciplinary collaboration among materials science, biology, and clinical medicine, metal ion assisted phototherapy is expected to move from a promising experimental concept toward a transformative therapeutic option for cancer patients.

## Ethics approval and consent to participate

Not applicable.

## Consent for publication

All authors of this work agreed to publish.

## Funding

The work was supported by 10.13039/501100001809National Natural Science Foundation of China (62205094), Zhejiang Medical Health Science and Technology Project (2024KY693), Excellent research start-up fund of 10.13039/501100015258Zhejiang Provincial People's Hospital (ZRY2021A002, ZRY2022J001), Adjunct Talent Fund of Zhejiang Provincial People’s Hospital.

## CRediT authorship contribution statement

**Yang Chen:** Conceptualization, Data curation, Investigation, Project administration, Software, Visualization, Writing – original draft, Writing – review & editing. **Yehui Kang:** Data curation, Formal analysis, Methodology, Resources, Validation, Writing – original draft. **Lichen Ji:** Formal analysis, Investigation, Resources, Writing – original draft. **Liya Yu:** Data curation, Project administration, Validation. **Longcai Liu:** Formal analysis, Methodology, Software. **Xiaozhou Mou:** Conceptualization, Formal analysis, Resources, Supervision, Writing – review & editing. **Yu Cai:** Conceptualization, Data curation, Funding acquisition, Project administration, Resources, Supervision, Writing – review & editing.

## Declaration of competing interest

The authors declare that they have no competing interests.

## Data Availability

No data was used for the research described in the article.

## List of abbreviations

photodynamic therapy: PDT
photothermal therapy: PTT
metal ion-dependent cell death: MIDCD
chemodynamic therapy: CDT
sonodynamic therapy: SDT
photosensitive substances: PSs
reactive oxygen species: ROS
lipid peroxidation: LPO
singlet oxygen: ^1^O_2_
immunogenic cell death: ICD
antigen-presenting cells: APCs
glutathione: GSH
transferrin receptor 1: TFR1
divalent metal transporter 1: DMT1
polyunsaturated fatty acids: PUFAs
mitochondrial permeability transition pore: MPTP
buthionine sulfoximine: BSO
tricarboxylic acid cycle: TCA
Elesclomol: ES
8-Hydroxyquinoline: 8HQ
Disulfiram: DSF
transient receptor potential vanilloid 1: TRPV1
curcumin: Cur
